# Genome-wide comparison between IL-17 and combined TNF-alpha/IL-17 induced genes in primary murine hepatocytes

**DOI:** 10.1186/1471-2164-11-226

**Published:** 2010-04-07

**Authors:** Titus Sparna, Julia Rétey, Kathrin Schmich, Ute Albrecht, Katrin Naumann, Norbert Gretz, Hans-Peter Fischer, Johannes G Bode, Irmgard Merfort

**Affiliations:** 1 Department of Pharmaceutical Biology and Biotechnology, University of Freiburg, D-79104 Freiburg, Germany; 2 Genedata AG, Maulbeerstrasse 46, CH-4016 Basel, Switzerland; 3 Department of Gastroenterology, Hepatology and Infectiology, Heinrich Heine University Düsseldorf, D-40225 Düsseldorf, Germany; 4 Medical Research Center, University Hospital Mannheim, D-68135 Mannheim, Germany

## Abstract

**Background:**

Cytokines such as TNF-alpha and IL-1beta are known for their contribution to inflammatory processes in liver. In contrast, the cytokine IL-17 has not yet been assigned a role in liver diseases. IL-17 can cooperate with TNF-alpha to induce a synergistic response on several target genes in different cell lines, but no data exist for primary hepatocytes. To enhance our knowledge on the impact of IL-17 alone and combined with TNF-alpha in primary murine hepatocytes a comprehensive microarray study was designed. IL-1beta was included as this cytokine is suggested to act in a similar manner as the combination of TNF-alpha and IL-17, especially with respect to its role in mRNA stabilization.

**Results:**

The present microarray analysis demonstrates that primary murine hepatocytes responded to IL-17 stimulation by upregulation of chemokines and genes, which are functionally responsible to increase and sustain inflammation. Cxcl2, Nfkbiz and Zc3h12a were strongly induced, whereas the majority of the genes were only very moderately up-regulated. Promoter analysis revealed involvement of NF-kappaB in the activation of many genes. Combined stimulation of TNF-alpha/IL-17 resulted in enhanced induction of gene expression, but significantly synergistic effects could be applied only to a few genes, such as Nfkbiz, Cxcl2, Zc3h12 and Steap4. Comparison of the gene expression profile obtained after stimulation of TNF-alpha/IL-17 versus IL-1beta proposed an "IL-1beta-like effect" of the latter cytokine combination. Moreover, evidence was provided that modulation of mRNA stability may be a major mechanism by which IL-17 regulates gene expression in primary hepatocytes. This assumption was exemplarily proven for Nfkbiz mRNA for the first time in hepatocytes. Our studies also suggest that RNA stability can partially be correlated to the existence of AU rich elements, but further mechanisms like the RNase activity of the up-regulated Zc3h12a have to be considered.

**Conclusions:**

Our microarray analysis gives new insights in IL-17 induced gene expression in primary hepatocytes highlighting the crosstalk with the NF-kappaB signaling pathway. Gene expression profile suggests IL-17 alone and in concert with TNF-alpha a role in sustaining liver inflammatory processes. IL-17 might exceed this function by RNA stabilization.

## Background

The liver is an important organ with high regenerative potential and complex functions. Regeneration occurs through growth factor- and cytokine-mediated proliferation of differentiated hepatocytes [1]. In the priming phase of liver regeneration induced by partial hepatectomy or mitogenic effects, TNF-α and IL-6 are involved, whereas IL-1β is known to be a potent inhibitor of hepatocyte proliferation. Overall, these cytokines are known for their proinflammatory properties contributing to inflammatory processes which also precede liver regeneration [1-4]. Recently, interleukin-17 (IL-17A) has stepped in the limelight as a further interesting proinflammatory cytokine produced predominantly by a distinct class of Th lymphocytes, the Th17 cells. IL-17 is thought to be important for host defence against bacterial infection and plays a significant role in local tissue inflammation through modulating cytokine and especially chemokine production by various cells. Evidence was provided that neutrophil recruitment is mediated through upregulation of chemokine expression on target cells like epithelial cells [5,6]. Interestingly, studies in patients with hepatocellular carcinoma revealed a higher number of IL-17 expressing lymphocytes in tumor tissue [7], which correlates with reduced survival [8], suggesting IL-17 a tumor promoting role. Moreover, IL-17 was demonstrated to be dramatically increased in patients with alcoholic liver disease [9], where it is discussed as a key feature important for liver neutrophil recruitment. However, IL-17 has not yet been assigned a role in liver regeneration. Altogether, its moderate influence on gene expression suggests that it rather sustains, than induces inflammatory processes [10]. Interestingly, blocking of IL-17 prior to systemic TNF-α application, protects mice from TNF-α induced systemic inflammatory response syndrome [11].

The latter observation points to the fact that IL-17 can cooperate with TNF-α to induce a synergistic response on several target genes which has been shown in various cell lines [12,13]. However, no data exist for primary hepatocytes or any hepatoma cell line in this respect, therefore further information is needed. Microarray data exist upon stimulation with IL-17 for human vascular smooth muscle cells [14], bronchial epithel cells [5,15], and upon TNF-α/IL-17 stimulation for the preosteoblast cell line MC3T-E1, ST2 and MEFs [12], human rheumatoid arthritis synoviocytes [13,16], MEFs and HeLa cell lines [17]. Strikingly, chemokines always belong to the most prominently expressed genes.

Almost all known IL-17 target genes are also regulated by IL-1β and TLR ligands, such as LPS, suggesting an overlap of parts of their signaling pathways. Computational analysis of 18 IL-17 target gene promoters revealed binding sites for the transcription factors NF-κB, CCAAT/enhancer-binding protein (C/EBP), AP1 and OCT1 as over-represented. However, joint occurrence of NF-κB and C/EBP is not a common feature of all IL-17 genes [12,18] Thus, genes with an immunoregulatory function possess binding sites for both, whereas promoters from chemokines do not commonly have C/EBP, AP1 or OCT1, but NF-κB sites [18]. IL-17 is somewhat different from classical NF-κB activators, as it induces a weak NF-κB DNA binding activity and surprisingly could not activate NF-κB reporter gene constructs transfected in several cell lines [6].

Furthermore, IL-17 is known to influence gene expression by modulating RNA stability [17] which can be correlated to the occurrence of AU-rich elements (ARE) mostly in the 3' untranslated region of the mRNA. These sequences represent interaction sites for stability modulating proteins like Zfp36 (encoding tristetraprolin) [6,19-21]. Although the underlying mechanism for the potent synergy of IL-17 with TNF-α is not well understood it is presumed that IL-17 is involved in enhancement of transcript stability, particularly in those genes containing AREs.

As no data exist for primary hepatocytes on the influence of IL-17 and its potent synergy with TNF-α, both very important proinflammatory cytokines, a study was designed to identify hepatocyte specific gene expression changes induced by IL-17 alone and in combination with TNF-α. Moreover, analyses were carried out to elucidate the possible impact of IL-17 on the modulation of mRNA stability. As the used cytokines belong to the group of pro-inflammatory mediators, the results obtained may increase our knowledge of the complex signaling network contributing to the development of inflammatory liver diseases.

## Results and Discussion

Primary hepatocytes were stimulated with TNF-α (2 ng/ml) alone or in combination with IL-17 (100 ng/ml) following the protocol of [12] for 1 and 4 h respectively, in order to observe changes in early as well as delayed gene expression profiles. For a detailed overview on the experimental setup please refer to Additional file 1, Table S1. Titration experiments revealed 100 ng/ml of recombinant IL-17 to be sufficient for maximal induction (data not shown). Both cytokine concentrations have been evaluated in advance by determining their capacity to induce mRNA expression levels of the hallmark target genes IL6, Nfkbiz and iNOS in response to different concentrations alone or in combination. We also included stimulation of primary hepatocytes with IL-1β in the same experimental approach. We chose IL-1β, because this cytokine might act in a similar way as the combination of TNF-α and IL-17 by initiating transcription via NF-κB as well as stabilizing short-lived mRNA possessing enhanced numbers of ARE [17]. Since previous observations revealed an ongoing cultural adaptation process accompanied with distinct changes in gene expression (Zellmer et al. unpublished data), gene profiles of untreated cells were recorded for both time points as time-matched references, a procedure different to conventional analyses.

**Figure 1:**
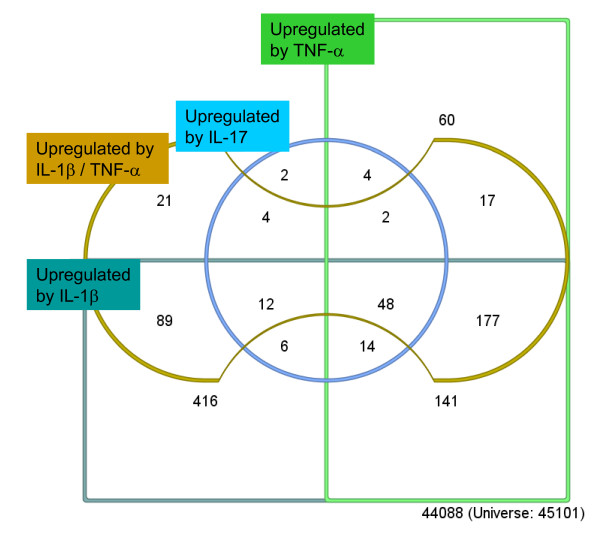
**Venn Diagram describing relationships between up-regulated probe sets transcripts after treatment of hepatocytes with different cytokines or combinations thereof**. Genes up-regulated by IL-1β (dark green; 903 probe sets, ST q-value < 0.00018) and TNF-α (green; 463 probe sets, ST q-value < 0.0014) are located within the two rectangles, genes up-regulated by IL-17 stimulation (blue: 92 probe sets, ST q-value < 0.012) in the centre circle and genes up-regulated by combined stimulation of TNF-α and IL-17 (brown, 370 probe sets, ST q-value < 0.0014) in the bone shaped structure.

A two-way analysis of variance for repeated measures (rANOVA) with factors time (1 h, 4 h) and treatment (control, IL1β, TNF-α, IL-17, TNF-α/IL-17) was performed to identify genes which are regulated by any of the investigated cytokines. 1631 probe sets (representing 1341 genes) were regulated (factor treatment, ST q-value < 0.045), 13357 probe sets were not significantly influenced and 30113 probe sets were excluded from the analysis because they contained low quality results (quality p-value ≥ 0.05 according to MAS5) in at least one of the experiments. To dissect which of the cytokines is responsible for the regulation of which genes, the 1631 regulated probe sets were subjected to further mathematical analysis. For each cytokine treatment a separate two-way rANOVA with factors time (1 h, 4 h) and treatment (control 1 h, 4 h, cytokine) was performed. The regulated genes were separated in two groups (up-regulated by cytokine, downregulated by cytokine) using a K-means cluster algorithm [22]. The results with the respective up-regulated genes are depicted in Figure 1 and Table 1. 903 probe sets were induced by IL-1β (ST q-value < 0.00018), 463 probe sets by TNF-α (ST q-value < 0.0014), 92 probe sets by IL-17 (ST q-value < 0.012) and 370 probe sets by the combination of TNF-α/IL-17 (ST q-value < 0.0014).

**Table 1:** Subgroups of up-regulated genes.

Group	Group Size (probe sets)	Group Size (Genes)	NF-κB target genes (Bioinf)	NF-κB target genes (Lit)	ARE
IL-17	2	2	-	-	-
TNF/IL-17	21	21	8 (38%)	1 (5%)	9 (43%)
IL1 + IL17	6	6	-	-	-
IL1 + TNF	141	135	23 (17%)	8 (6%)	24 (18%)
IL1 + TNF/IL17	89	80	9 (11%)	18 (23%)	15 (19%)
IL-17 + TNF/IL17	4	4	-	-	-
IL17 + TNF	4	4	-	-	-
TNF + TNF/IL17	17	16	-	-	-
TNF + IL1 + TNF/IL17	177	151	39 (26%)	35 (23%)	25 (17%)
TNF + IL17 + TNF/IL17	2	2	-	-	-
IL1 + IL17 + TNF/IL17	12	10	-	-	-
TNF + IL1 + IL17	14	14	-	-	-
Up-regulated by all stimuli (TNF/IL-17, IL-17, TNF, IL-1)	48	40	23 (58%)	21 (53%)	15 (38%)

All expressed genes^a^	14.988	9.828	1369(14%)	311 (3%)	2076 (21%)

Probe sets and gene numbers are indicated as well as frequency of NF-κB transcription factor binding site (separated for the bioinformatic and literature based approach) and the frequency of AU rich elements (ARE). Groups with less than 20 genes have been excluded from the analysis.

^a ^This group comprises all genes which show at least one valid expression value

### IL-17 induced gene expression

At first genes up-regulated by stimulation with IL-17 in primary hepatocytes (92 probe sets representing 79 genes, Figure 1, blue circle, Additional file 2, Table S2) were further investigated. As expected for an inflammatory cytokine genes related to immune response and inflammation as well as several chemokines were found among the most strongly up-regulated ones. Accordingly, the search for overrepresented GO terms revealed "inflammatory response" as most significant (Fisher's Exact test, p-value = 1.5E-5) confirming previous observations in murine embryonic fibroblasts and osteoblasts [12]. In line with the observations reported by [12] the data presented herein indicate that in primary hepatocytes IL-17 itself is a weak inductor of gene expression compared to cytokines, such as TNF-α. This was similar with our study in primary hepatocytes, as only 11 probe sets (representing 8 genes) reached a fold induction of >2 fold at 1 h and 18 probe sets (representing 13 genes) at 4 h by IL-17 treatment. In contrast, in rheumatoid arthritis synoviocytes a microarray study revealed about 600 genes significantly regulated by two IL-17 isoforms after 12 h of stimulation [13].

**Figure 2:**
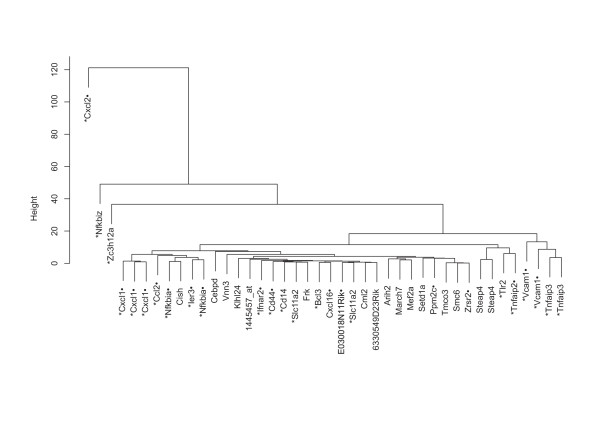
**Hierarchical clustering of 35 genes up-regulated by IL-17 in primary hepatocytes**. Two way rANOVA analyses were performed to identify genes up-regulated by IL-17. Genes with enhanced differential gene regulation between different cytokine stimulations were identified by filtering for a minimum SD >0.4. 42 out of the 92 probe sets identified by ANOVA remained and are presented in the dendrogram. NF-κB target genes are indicated by a star (*) for literature derived annotation or a dot (.) for bioinformatical annotation next to the gene symbol (for building the NF-κB lists refer to Additional file 1).

To focus on genes with substantial differential regulatory response towards IL-17, only probe sets reaching a standard deviation (SD) >0.4 in their fold induction ratios (measured over all experiments) were hierarchically clustered (42 probe sets = 35 genes) (see Figure 2). As NF-κB plays a crucial role for transcriptional regulation of genes expressed in the course of an inflammatory process, these genes were analyzed for NF-κB binding sites. Based on a literature or a bioinformatics approach several of the genes analyzed were identified as NF-κB target genes (see Figure 2, details on the generation of the lists can be found in Additional file 1).

As depicted in Figure 2 three genes of the cluster, namely Cxcl2 (also termed as MIP-2), NFkbiz and Zc3h12a, possessed unique regulatory properties, while the majority exhibits more homogeneously regulated expression profiles. The proinflammatory chemokine Cxcl2, which orchestrates neutrophil recruitment to inflammatory foci is known to be strongly induced by IL-1β [23] and to comprise a NF-κB binding site within its 5' regulatory region [24], but is only weakly up-regulated by IL-17 (1 h stimulation: 3.9 fold, 4 h: 4.9 fold upregulation). Contrariwise, the immediate early gene Nfkbiz (IκBzeta), which is also known to be strongly induced by IL-1β [25] is also significantly upregulated by stimulation with IL-17 for 1 h (p = 1 h: 11.0 fold, 4 h: 8.3 fold), whereas Zc3h12a (= Monocyte chemotactic protein-1 induced protein, MCPIP-1) is strongly up-regulated by IL-17 and this even surmounts induction by IL-1β, when assessed after 4 h stimulation (1 h: 6.4 fold, 4 h: 15.3 fold). Accordingly, the genes Zc3h12a (p-value = 2.8E-7, ST q-value = 4.9E-5) and Nfkbiz (p-value = 3.6E-5, ST q-value = 2.2E-3) show the highest p-values for IL-17 dependent gene expression in the rANOVA. Nfkbiz is a well known IL-17 target gene [12,26], whereas Zc3h12a has not yet been described as IL-17 dependent gene, but as NF-κB regulated [27]. Interestingly, both genes also display properties of a transcription factor [28-30].

When analyzing the remaining genes three subgroups can be distinguished from which two of them contain genes characterized in literature as immediately early genes (IEG) [31,32]. These genes are considered to require no *de novo *protein synthesis for upregulation of expression [33], presumably due to the regulation of preexisting transcription factors [33,34] and alleviated chromosomal accessibility [35]. The first group consists of the chemokines Cxcl1, Ccl2 as well as the transcription factors/inhibitors Nfkbia (IκBα), Cish and Ier3. Except for Cish, a transcription factor belonging to the SOCS-family, all genes are known and predicted as NF-κB driven genes, suggesting that NF-κB is the major transcriptional regulator of this group. The second subgroup contains genes encoding for the adhesion molecule Vcam1 and the NF-κB inhibitor Tnfaip3 (A20), which are also thought to be NF-κB target genes. Notably, IκBα and A20 are important negative feedback regulators of NF-κB signaling and are crucial for damping the proinflammatory activity. Except of Ccl2 all genes in these two groups contain AREs. The third subgroup includes Tlr2, Tnfaip2 (B94), both known as NF-kB target genes, and Steap4.

To analyze if IL-17 up-regulated genes are regulated by RNA stability modulation via AREs in primary hepatocytes, the 42 probe sets were screened for presence of AREs using the ARES3 database [36]http://brp.kfshrc.edu.sa/ARED/ (see Figure 2). From these probes ten genes could be annotated as ARE comprising genes. Interestingly, Nfkbiz was not annotated by the database, but was experimentally shown to be regulated by RNA stabilization related to the 3'UTR [37]. However, AREs do not preferentially occur in the group of highly regulated IL-17 target genes, but rather seem to be a common feature within the gene sets up-regulated by IL-17, as a comparable fraction of ARE annotated genes was identified for all 92 probe sets (81 genes) by the rANOVA.

Besides its impact on transcript stability of those genes harbouring AREs within their non-coding regulatory regions, IL-17 may also be involved in an alternative mechanism by which RNA stability is controlled. Thus, studies suggest, that the transcript stability of IL-17 target genes, such as Nfkbiz and Cxcl1, is ARE-independently regulated by alternative regulatory sequences located in the 3'UTR of these genes [38,39]. This could be particularly due to marked IL-17-mediated upregulation of the RNase Zc3h12a, which was shown to be responsible for the RNA decay of a subset of proinflammatory mediators comprising genes such as IL-6 [40]. This participation in IL-6 regulation places Zc3h12a into a central role for liver regeneration and liver repair as IL-6 is of critical relevance for an undisturbed course of these processes.

The rANOVA also revealed 126 probe sets which were downregulated by IL-17 treatment. Here, most genes show weak expression changes when compared to control. When applying the SD >0.4 filter only 17 genes remained, including Nalp12, a gene suppressing inflammatory responses in monocytes, Dusp22, which enhances IL-6 induced STAT3 dependent transcription, when downregulated [41], and connective tissue growth factor (Ctgf), which inhibits adipocyte differentiation [42]. The observed downregulation of immunosuppressive genes is in accordance with the primarily proinflammatory function of IL-17. When annotating the genes for NF-κB TFBS, a lower number of NF-κB target genes (10.3% (Fisher's Exact Test: p-value = 0.88)) were detected compared to the fraction of the up-regulated genes (29.1% (Fisher's Exact Test: p-value = 0.000013)) (based on the bioinformatical promoter analysis approach, described in Methods). Using the literature based list only 2% of the downregulated cluster compared to 23% of the up-regulated genes were annotated to contain NF-κB TFBS (Data in Additional file 2, Table S2). Promoter analysis revealed overrepresentation for 3 NF-κB related PFMs (V$NFKB65_01, p-Value > 7.88E-05, V$NFKB_Q6 p-value > 0.0006, MA0107, p-value > 0.001) and for an AP1 PFM (MA0003, p-value > 0.004).

### IL-17 induced genes in relation to the cytokines TNF-α and IL-1β

In order to gain an overview on gene expression in primary hepatocytes induced by the combination of TNF-α and IL-17 and to analyze possible relationships between TNF-α and IL-1β induced gene expression, treatment effect of the single cytokines and the combination of TNF-α/IL-17 was studied. In the following at first genes up-regulated by IL-17 are compared with that ones induced by the combination of IL-17/TNF-α and subsequently subgroups with genes up-regulated by various stimuli.

#### IL-17 induced genes in relation to IL-17/TNF-α

Direct comparison between the set of genes induced by IL-17 with that one up-regulated by the combination of IL-17/TNF-α, identifies similar gene-sets, while the cytokine-combination induces the majority to a higher degree (see Additional file 3, Table S3). To identify synergistically induced genes, the cooperative index as described by [13] was calculated. Among all significantly up-regulated genes, only Nfkbiz and Cxcl2 genes turned out to be synergistically induced after 1 h, and three genes (Cxcl2, Zc3h12a and Steap4) after 4 h (Additional file 4, Table S4). Additionally, Gm1960 which encodes for Cxcl3, a chemokine similar to Cxcl2, was also detected to be synergistically up-regulated at 4 h [43]. This small number of genes indicates, that in the case of TNF-α and IL-17 synergistic induction may not play a prominent role in gene expression in primary hepatocytes. This differs from those data reported for synoviocytes isolated from patients with rheumatoid arthritis where in total 130 genes were detected to be synergistically regulated after 12 h of stimulation [13].

#### Genes significantly up-regulated by IL-1β, TNF-α, IL-17 and IL-17/TNF-α

41 genes (48 probe sets) were identified to be up-regulated by all stimuli representing a gene group which is commonly up-regulated in hepatocytes in different inflammatory responses. Accordingly, GO analysis revealed "inflammatory response" as overrepresented term with a p-value < 10-6 (Additional file 5, Table S5) and 53% of the genes were confirmed or predicted as NF-κB target genes (Table 1). The importance of NF-κB is underlined by the fact that the most significantly up-regulated TFBS are assigned to position frequency matrices of different NF-κB subunits or heterodimers (Additional file 6, Table S6).

**Figure 3:**
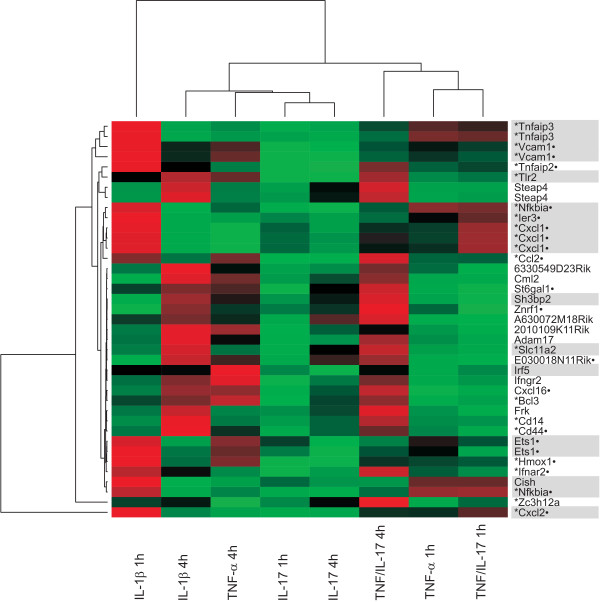
**Two dimensional clustering of transcripts up-regulated by IL-1β, TNF-α, IL-17 and the combination of TNF-α/IL17 ("all up"), filtered SD >0.4**. Genes are mainly distinguishable whether they are up-regulated at an early (1 h) or at a late time point (4 h). Note that genes up-regulated after 4 h of IL-1β stimulation are generally also up-regulated at 4 h stimulation with TNF-α/IL-17. Transcripts containing ARE sites are highlighted in grey.

To get a more clearly structured picture probe sets were filtered for SD >0.4 and subjected to hierarchical clustering. Expression ratios are presented as heat map (see Figure 3). Interestingly, several genes up-regulated by IL-1β at 4 h showed a similar expression as genes induced by TNF-α/IL-17 at 4 h (see also Additional file 3, S3). This correlation was not observed for genes already up-regulated after 1 h of IL-1β treatment. To evaluate if IL-17 in addition to TNF-α will cause a shift towards a more "IL-1β like" gene expression, the Pearson correlation coefficient was calculated between IL-1β (4 h) and TNF-α (4 h) or the combination of TNF-α/IL-17 (4 h), respectively (based on the fold induction ratios of the 48 probe sets) [44]. A coefficient of 0.69 was calculated for TNF-α versus IL-1β and increased to 0.92 by costimulation with IL-17/TNF-α (see Additional file 7, Figure S1). IL-17 alone was not sufficient for the increased correlation observed (see Additional file 7, Figure S1B). Interestingly, when all probe sets of the chip were taken into account a slightly lower coefficient of 0.79 was obtained between IL-1β and IL-17/TNF-α (see Additional File 7, Figure S3). Thus, it can be assumed that in the presence of IL-17 a subset of genes up-regulated by TNF-α may shift the expression profile towards "IL-1β like" properties [17]. This shift seems to be rather due to additional than synergistical effects.

Looking for common features within the subset of these 48 genes two groups can be distinguished: Immediate early type genes displaying characteristic high expression values for IL-1β 1 h data points (e.g. Cxcl2, Tnfaip3, Ier3) and delayed genes (e.g. Cd14, Cd44, Steap4), which are up-regulated just after 4 h of IL-1β stimulation.

Interestingly, Zc3h12a was markedly up-regulated (>30 fold after 4 h TNF-α/IL-17 stimulation), whereas Zfp36 encoding for tristetraprolin, one of the best understood RNA stability modulating factors in liver and a close family member to Zc3h12a, was only weakly induced (<2 fold for all stimulations). In case of Zfp36 its degrading effects on RNA are well known [45-47] whereas this property has been reported for Zc3h12a only recently [40]. The fact, that Zfp36 and Zc3h12a belong to the same family and possess structural similarity, but have different target mRNAs shows that regulation of RNA stability occurs in a comprehensive network. It therefore can be speculated that Zc3h12a represents a mechanism for IL-17 mediated differential gene expression. Since these findings assume Zc3h12a to be of special interest in hepatocytes we performed a detailed qRT-PCR analysis of RNA induction by the different stimuli (see Additional file 8, Figure S4).

#### Genes significantly up-regulated by TNF-α/IL-17 and IL-1β stimulation, but not by IL-17 or TNF-α alone

When analyzing the group comprising genes up-regulated after stimulation by TNF-α/IL-17 and IL-1β (89 probe sets mapping to 77 genes) similarities with the subset of the 48 probe sets up-regulated by all stimuli (section above) were observed. The fold increases were only moderate for all genes, since most strongly up-regulated genes were located in the "up-regulated by all stimuli" subset. Again, the Pearson correlation coefficient was calculated for these probe sets and a coefficient of 0.27 was obtained for the 4 h stimulated probe sets (IL-1β versus TNF-α), which increased, as expected, to 0.92 when IL-1β was correlated versus IL-17/TNF-α (see Additional file 7, Figure S2). Hence, the hypothesis of the "IL-1β like effect" for TNF-α combined with IL-17 might also be applied for this gene group. It seems that the underlying mechanism needs some time to fully modulate gene expression, since the correlation after 1 h of stimulation was significantly lower (0.61, IL-1β versus IL-17/TNF-α, data not shown).

GO analysis did not show an overrepresentation of inflammatory response for this group of genes (Fisher's Exact Test: p > 0.2). Instead, nucleocytoplasmic transport (p < 0.0005), cell death (p < 0.0008) and acute phase response (p < 0.003) were among the most highly over-represented biological processes (See Additional file 5, Table S7). Promoter analysis revealed AP-2 as the top over-represented TFBS (p < 5.9E-5). None of the NF-κB PFMs was significantly over-represented (p > 0.05 for all NF-κB PFMs).

Further cluster analysis (filter SD >0.4) showed only one characteristic subgroup with high expression after TNF-α/IL-17 and IL-1β stimulation at 4 h (top rows in Additional file 9, Figure S5). This gene set included genes related to acute phase response, such as hepcidin (Hamp1), Oncostatin M receptor (Osmr), suppressor of cytokine signaling (Socs3), and serum amyloid A, but altogether, only a restricted number of acute phase proteins were detected, an observation which may be due to the time window used.

Another gene of this subgroup which might be of interest is Tnfrsf1b encoding for tumor necrosis factor receptor superfamily member1b since it may indicate a positive regulatory loop. This may in part contribute to the potentiating effect of IL-17/TNF-α as already suggested for TNFRII in human rheumatoid arthritis synoviocytes [13]. However, this regulatory effect should become more obvious at later time points which have not been studied here.

#### IL-1β, IL-17, and TNF-α/IL-17, but not TNF-α up-regulated genes with Nfkbiz as prominent representative

Genes of this group (12 probe sets representing 9 genes) are closely related to the previous group and are characterized by a weak TNF-α inducibility. Cluster analysis revealed as the most prominent member with significant up-regulated gene expression Nfkbiz (encoding IκBzeta), the gene mentioned above (see Additional file 10, Figure S6 and Table 1). Nfkbiz was also shown to be mainly up-regulated by IL-1β, but not by TNF-α [48]. Further genes include the transcription factors Cebpd, Vanin3 (Vnn3), Rho family GTPase 3 (Rnd3) and Adamts1.

Nfkbiz can function as transcription factor being also involved in nucleosomal opening thereby directly influencing target gene accessibility and regulation [49]. Nfkbiz is also reported to act in a cofactor like manner by interacting with p50/p65 or p50 homodimers [30,50-52]. Recently, a correlation was demonstrated to exist between Nfkbiz and the transcription factor C/EBPδ (CCAAT/enhancer binding protein) in MEF cells [53]. Additionally, Nfkbiz and C/EBPs were proven to induce a subset of genes by cooperating with one another in HEK293 cells and osteoblasts derived cells [54,55]. Among the 9 up-regulated target genes is Cebpd, which in turn is required for *de novo *translation of Nfkbiz. To which extend this feed forward loop may influence target gene expression in hepatocytes and which consequences may result from Nfkbiz dependent gene regulation has to be clarified in future studies.

### TNF-α and IL-17 synergistically induce Nfkbiz mRNA expression independently of NF-κB

**Figure 4:**
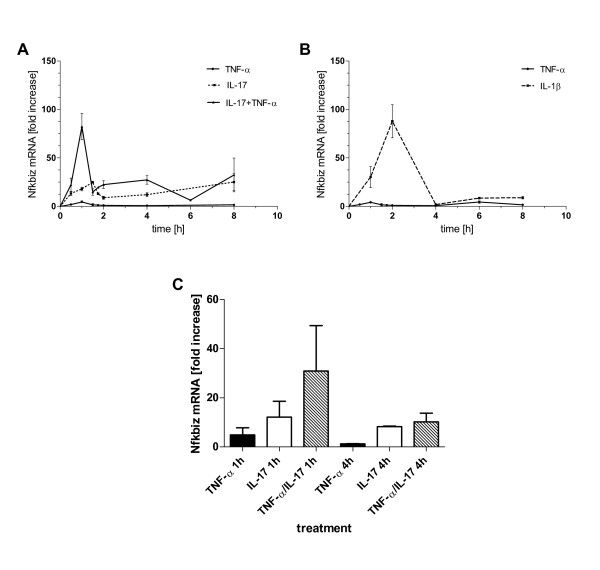
**Time course of Nfkbiz mRNA expression**. Primary hepatocytes were treated with TNF-α (2 ng/ml), IL-17 (100 ng/ml) or the combination of both (A) or with IL-1β (20 ng/ml) (B) and Nfkbiz mRNA expression was determined by qRT-PCR after the indicated times. Data represent means +/- SEM of at least three independent experiments. Nfkbiz mRNA expression levels from the microarray experiment for comparison are shown in (C).

To verify the expression data of Nfkbiz obtained by the microarray, qRT-PCR analysis were performed. Primary hepatocytes were treated with TNF-α, IL-17 or the combination of both for different times up to 8 h and subsequently Nfkbiz mRNA levels were measured. Figure 4A confirms for hepatocytes that TNF-α alone results in a very weak induction of Nfkbiz gene expression whereas IL-17 leads to an increased expression with a peak at 1.5 h and 8 h. Strikingly, IL-17 in combination with TNF-α leads to a synergistical increase in transcript expression. mRNA levels remain elevated up to 4 h and show a second induction phase at 8 h. This is consistent with our microarray data (see Table 2 and Figure 4C). Furthermore, IL-1β induced Nfkbiz mRNA expression was verified and compared to the other stimuli. Figure 4B shows a similar kinetic induced by IL-1β as it has been shown for TNF-α and IL-17. Note that microarray analyses have been performed at 1 and 4 hours and therefore the maximum peak might not have been detected at these time points. Taken together qRT-PCR data could nicely verify the microarray data.

To test whether synergistic activation of transcription factor NF-κB is involved in IL-17/TNF-α induced upregulation of Nfkbiz, we performed NF-κB EMSAs with nuclear extracts of primary mouse hepatocytes. NF-κB DNA binding was markedly increased after treatment with TNF-α but only very slightly after IL-17 treatment. Interestingly, cells stimulated with the combination of both cytokines showed no increase in NF-κB DNA binding compared to cells treated with TNF-α alone (Data not shown)

These findings are in agreement with previous reports where the combination of IL-17/TNF-α did not induce a greater transcriptional initiation than TNF-α alone on CXCL1 expression in MEF 3T3 cells [17] suggesting that IL-17 mediates its effects on gene expression on the level of transcript stability.

### IL-17 increases the t1/2 of Nfkbiz mRNA

**Figure 5:**
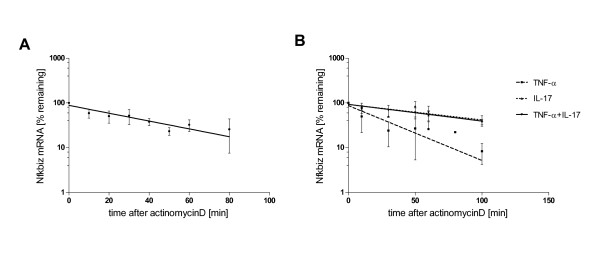
**IL-17 increases the half life time of Nfkbiz mRNA by modulating RNA stability**. (A) Cells were incubated with actinomycin D (5 μg/ml) to stop further transcription and Nfkbiz mRNA levels were determined after the indicated times. To calculate the RNA half life time (34.36 minutes) results were expressed as % remaining RNA over time and one phase decay curves were applied. (B) Influence of cytokines was tested by incubation of primary hepatocytes with IL-17 (100 ng/ml) for 30 minutes with or without additional TNF-α (2 ng/ml) stimulation or TNF-α alone. Then actinomycin D was added and cells were further incubated for the indicated times. Nfkbiz mRNA levels were determined and plotted and analyzed as described above. RNA half life time was calculated and shows a remarkable increase up to 87.26 minutes induced by IL-17 and 80.47 minutes by IL-17/TNF-α compared to 24.56 minutes induced by TNF-α treatment alone. Data represent means +/- SD of at least three independent experiments.

To determine the effect of IL-17 and TNF-α on mRNA stabilization we first measured the half life time of Nfkbiz RNA itself. Figure 5A shows the decay of Nfkbiz RNA over time and reveals a half life time of 34.36 minutes calculated by one phase decay non-linear regression.

Then the ability of the two cytokines and their combination to stabilize Nfkbiz mRNA was tested. Therefore, primary hepatocytes were incubated with IL-17 and/or TNF-α for 30 minutes, subsequently actinomycin D was added to block further transcription. After incubation for the indicated times Nfkbiz mRNA levels were determined and plotted against time. Figure 5B clearly shows that TNF-α alone is not able to significantly modulate the t_1/2 _of Nfkbiz mRNA (24.56 minutes after TNF-α treatment). In contrast, IL-17 prolongs the half life time up to 87.26 minutes and the combination of IL-17/TNF-α also stabilized Nfkbiz mRNA and resulted in a t_1/2 _of 80.47 minutes. These findings indicate that IL-17 is able to stabilize Nfkbiz transcripts and prolongs the mRNA half life time to more than the double of its original time. Thus, we could demonstrate for the first time in hepatocytes that IL-17 treatment enhances Nfkbiz mRNA stability. Previously, this was shown for several genes in MEFS [17] and for Nfkbiz in NIH3T3 cells [37]. Importantly, the conclusion that enhancement of mRNA stability may be a major mechanism by which IL-17 regulate gene expression can also be applied to primary hepatocytes.

## Conclusions

The present microarray analysis demonstrates for the first time that primary murine hepatocytes are also sensitive to short term IL-17 stimulation corroborating the assumption that hepatocytes also participate and are functionally influenced in the context of an inflammatory response. Hepatocytes show a rather low induction of gene expression level, as it is also observed for fibroblasts, muscle and epithelial cells, whereas rheumatoid arthritis synoviocytes show upregulation of a higher number of genes following IL-17 stimulation [13]. In agreement with the literature [12] combined stimulation of TNF-α/IL-17 resulted in enhanced induction of gene expression, but significantly synergistic effects could be applied only to few genes, especially to chemokines. Here, gene expression of Cxcl1 and Cxcl2 deserves special interest because of their function to attract neutrophils and lymphocytes thereby contributing to inflammatory processes and to the control of the composition of the immune competent cells recruited [6]. However, a substantial enhancement of pro-inflammatory genes was observed providing additional evidence that IL-17 may play an important role in supporting and sustaining inflammatory conditions including inflammatory liver diseases.

Promoter analysis unambiguously revealed that NF-κB is involved in the activation of many IL-17 target genes related to inflammation, especially in that ones with a high expression level. Although C/EBP is also described to have binding sites in close proximity to NF-κB TFBS, promoter analysis did not reveal its significant overrepresentation. However, Cebpd was found among the 9 most up-regulated genes following 4 h of TNF-α/IL-17 combined stimulation. This discrepancy may be explained that C/EBP regulated genes are not yet up-regulated at the chosen time point in our microarray analysis.

One of the most up-regulated genes turned out to be Nfkbiz (encoding IκBzeta) which was only slightly induced following TNF-α stimulation, but reached nearly the level observed for IL-1β after costimulation with IL-17/TNF-α. IκBζ can function as transcription factor and is involved in gene accessibility [49]. Therefore, it can be assumed that Nfkbiz may play an important role in fine tuning proinflammatory responses and influencing the downregulation and duration of inflammatory reactions in hepatocytes. Moreover, Nfkbiz when deregulated may even pave the way for malignancy, which may in part explain the oncogenic potential of IL-17 [56].

Our literature and bioinformatics based approach provides evidence that modulation of mRNA stability may also be a major mechanism by which IL-17 regulates gene expression in primary hepatocytes. Using Nfkbiz as prominent example this assumption was experimentally confirmed for the first time in hepatocytes. RNA stability can partly be correlated to the existence and number of ARE [31]. This is obviously the case when genes are highly up-regulated, such as chemokines. However, alternative proteins may be involved, as mRNA of Nfkbiz was shown to be controlled by a sequence located in the 3' untranslated region of the mRNA independently from the also present AREs [39]. A prominent candidate being involved in mRNA stability may be the RNase Zc3h12a which was shown here to be highly induced in primary hepatocytes and which is reported to be responsible for destabilization of the IL-6 mRNA in macrophages [40]. Interestingly, tristetraproline (Zfp36), one of the best understood RNA modulating factors in liver and a close family member to Zc3h12a seems not to play a significant role in the time window examined, since its RNA was not significantly induced. Altogether, our microarray analysis gives new insights how IL-17 may contribute to early inflammatory events in hepatocytes underlining again the importance of this cytokine in inflammatory diseases, and especially in inflammatory and autoimmune diseases of the liver.

## Methods

### Isolation and cultivation of primary hepatocytes

Primary hepatocytes from six mice were prepared and cells from two mice were pooled, respectively, seeded in collagen coated six well plates and cultured according to SOP reported by [57]. Each set of pooled cells were treated with IL-1beta (1 or 4 h), TNF-alpha (1 or 4 h), IL-17 (1.5 and 4.5 h) or IL-17 (0.5 h) followed by TNF-α (1 h or 4 h). Thus, three biological replicates were performed. Details of experimental conditions, stimulation and an experimental design are described in Additional file 1.

Hepatocyte isolation from these mice was approved by the animal experimental committees and animals were handled and housed according to specific pathogen free (SPF) conditions.

### RNA preparation

RNA preparation was based on anion exchange chromatography (RNEasy, Qiagen) as described in Additional file 1.

### Target labeling, hybridization and microarray processing

Performance was done according to the manufacturer's directions (Affymetrix Inc.) by the Microarray Core Facility, University Clinic, Mannheim. The mouse whole genome 430 V2 Affymetrix microarray was used for hybridization. Details of the procedure are available from the Microarray Core Facility web-site at http://www.ma.uni-heidelberg.de/inst/zmf/affymetrix/.

### mRNA stability

To determine the half life time of Nfkbiz mRNA, cells were incubated with actinomycin D (5 μg/ml, Alexis) for the indicated times to inhibit further transcription. Then total RNA was extracted and Nfkbiz mRNA expression quantified by qRT-PCR. Results are presented as % remaining mRNA compared to mRNA levels of untreated cells. To measure mRNA stability in response to cytokine treatment cells were stimulated with TNF-α (2 ng/ml), IL-17 (100 ng/ml) or the combination of both in the presence of actinomycin D whereas IL-17 was preincubated for 30 minutes. Nfkbiz mRNA expression was again quantified by qRT-PCR after the indicated times and relative mRNA abundance was calculated by setting the values of the Nfkbiz mRNA levels induced by 30 minutes IL-17 preincubation to 100%. Percentages were plotted against time, one phase decay curves were calculated (GraphPad Prism 5) and the time to 50% mRNA decay was deduced. Means of three independent experiments are presented.

### Expression data processing

Mouse430V2 Affymetrix CEL data files were uploaded into Expressionist Refiner Array^® ^version 4.5 (Genedata AG, Basel, Switzerland) for data condensation. Oligo hybridization intensities were condensed to probe set expression values using the MAS5 Affymetrix Statistical algorithm according to [58]. Condensed data were normalized using Expressionist Analyst^® ^version 4.5 (Genedata AG, Basel, Switzerland). Absolute probe set expression values were scaled on the chip level by logarithmic mean normalization (for each chip, the logarithmic mean of all probe set values was scaled to 10000). Hierarchical clustering of experiments revealed systematic expression differences for the three biological replicates of the mouse hepatocyte cultures (= factor series). To compensate for these systematic differences, data from each of the three replicate series were separately standardized on the probe set level by point-wise division (for each probe set and series, the median of the probe set values of all experimental conditions (Control, IL-17, TNF-α, IL-17/TNF-α, IL-1β) was standardized to 1). Data points with a quality p-value > = 0.05 according to MAS5 were excluded from further analyses. The expression data are publically available in the NCBI GEO database under series accession GSE19272 http://www.ncbi.nlm.nih.gov/geo/.

### Expression data analyses

Two-way analyses of variance for repeated measures (rANOVA) were performed in Expressionist Analyst to identify differentially expressed probe sets (factors treatment and time; probe sets with missing values with respect to the experiment scope of the respective ANOVA were excluded from the analysis). The significance level was set at α < 0.01. The corresponding false discovery rates (FDR, Storey-Tibshirani (ST) q-values, [59] are reported in the result section. Probe sets jointly regulated by two or more cytokine treatments were identified by intersecting groups of regulated genes determined by the ANOVAs. Fold factors were calculated from the geometric means of the three biological replicates of each condition versus the time matched control.

### Promoter analysis

Transcription factor binding sites (TFBS) were predicted for murine (NCBI mouse genome version 33) promoter regions (-5000 to +500 bp with respect to the transcription start site) on a genome-wide scale using Genedata Phylosopher^® ^version 6.5 Promoter Explorer (PE). PE predicts TFBSs based on a phylogenetic footprinting approach using normalised position frequency matrices (PFM) by three filtering steps. (1) Identification of conserved subregions within orthologous promoters of phylogenetically related genomes (NCBI mouse genome version 33 vs. NCBI human genome version 34). The conservation threshold was set to 70% identity within 50 bp sliding windows. (2) Identification of candidate binding sites by screening the conserved promoter subregions for consensus with PFMs (using default settings). PFMs were derived from TRANSFAC^® ^(version 3.2, [60,61]. (3) Further filtering of candidate binding sites for significantly enhanced consensus as compared to random DNA sequences having the same nucleotide fractions (A, T, G and C content) as the respective promoter region (bootstrapping p-value < 0.0001).

### List of NF-κB target genes

NF-κB target genes were identified as described in Additional file 1.

### Biological context analyses

Over-representation of biological processes [62]; http://www.geneontology.org and of TFBSs (based on TFBS predictions as described in section Promoter analysis) in gene groups of interest was estimated by Fishers' Exact tests using Expressionist Analyst. The mapping from probe sets to genes was performed using the Phylosopher seqmap tool based on the NCBI mouse genome version 33.

## Abbreviations

ARE: AU-rich elements; FDR: false discovery rate; IEG: Immediate early genes; qRT-PCR: quantitative real time PCR; TFBS: Transcription factor binding site(s); UTR: Untranslated region; GO: Gene ontology.

## Authors' contributions

TIS, JUR, KAS; UTA, HPF, JOB and IRM were involved in the study design. TIS analysed the results and provided the first draft. JUR performed condensation and normalisation of the microarray data, the N-way ANOVAs as well as the GO- and promoter analyses. KAS carried out a large part of the experiments, wrote a part of the manuscript and contributed valuable discussions. UTA and KAN have done some experiments. NOG was involved in performing the microarray analyses. JOB contributed valuable discussions and corrections. IRM supervised, promoted and coordinated the studies and wrote the manuscript. All authors read and approved the final manuscript.

## Supplementary Material

Additional file 1 **Additional information on methods and experimental design**. Additional information on experimental design and methods for hepatocyte isolation and cultivation, RNA preparation, qRT-PCR and preparation of the NF-κ target gene lists. Contains Table S1: Experimental design of the microarray analysis. Click here for file

Additional file 2 **Fold change values for all IL-17 significantly regulated genes**. Table S2: Genes identified as upregulated and downregulated following IL-17 stimulation. Click here for file

Additional file 3 **Genes up-regulated by combined TNF-α and IL-17 stimulation**. Table S3: Genes up-regulated by combined TNF-α and IL-17 stimulation. Click here for file

Additional file 4 **Genes showing synergistic or inhibitory regulation following combined TNF-α/IL-17 treatment**. Table S4: Genes showing synergistic/inhibitory regulation following combined TNF-α/IL-17 treatment. Click here for file

Additional file 5 **Gene onthology analysis**. Over-representation of GO biological processes in genes upregulated by all stimuli (Table S5) or the subgroup upregulated by IL-1β and TNF-α/IL-17 (Table S7). Click here for file

Additional file 6 **TFBS-overrepresentation analysis**. Table S6: Over-representation of transcription factor binding sites in promoters of genes upregulated by all stimuli. Click here for file

Additional file 7 **Calculation of the Pearson correlation coefficient to study the "IL-1β like" effect in different gene subsets**. Calculation of the Pearson correlation coefficient reveals a shift towards "IL-1β " like expression following combined TNF-α/IL-17 treatment. Contains additional figures: Figure S1: Calculation of the Pearson correlation coefficient to study the "IL-1β like" effect for the 48 probe sets group. Figure S2: Calculation of the Pearson correlation coefficient to study the "IL-1β like" effect for the 89 probe sets group. Figure S3: Calculation of the Pearson correlation coefficient to study the "IL-1β like" effect for those probe sets for which log-ratio data could be calculated (6039 from 14988 probe sets). Click here for file

Additional file 8 **Time resolved gene expression of Zc3h12a**. Figure S4: Time-course of Zc3h12a mRNA expression. Click here for file

Additional file 9 **Heatmap of genes upregulated by IL-1β and the combination TNF-α/IL-17**. Figure S5: Heatmap of the genes up-regulated by IL-1β and the combination of TNF-α and IL-17 (89 probe sets). Click here for file

Additional file 10 **Hierarchical cluster analysis of genes upregulated by IL-1β, IL-17 and TNF-α/IL-17**. Figure S6: Hierarchical cluster analysis of the probe sets (12) upregulated by IL-1β, IL-17, and TNF-α/IL-17, but not TNF-α alone. Click here for file
