# Supplementary material for: Genome-wide comparison between IL-17 and combined TNF-alpha/IL-17 induced genes in primary murine hepatocytes
Source: BMC Genomics. 2010 Apr 7;11:226. doi: 10.1186/1471-2164-11-226 (PMC2858152; doi:10.1186/1471-2164-11-226)
Supplement: Additional file 1 — Additional information on methods and experimental design. Additional information on experimental design and methods for hepatocyte isolation and cultivation, RNA preparation, qRT-PCR and preparation of the NF-κ target gene lists. Contains Table S1: Experimental design of the microarray analysis. [file 1471-2164-11-226-S1.PDF]

Additional File 1: Additional information on methods and experimental design

Isolation and cultivation of primary hepatocytes:

Hepatocytes were isolated from the liver of male BL6 mice by collagenase perfusion as described before [1], washed and allowed to attach to collagen coated 6 well plates (1 Mio cells/well) in Williams E medium supplemented with 100 nmol/l dexamethasone and 1 g/ml insulin. Cell viability was tested by trypan blue exclusion and exceeded generally 87%. In order to reach the necessary amount of cells, hepatocytes from two mice were pooled prior to seeding. Following 4h of adherence phase medium was exchanged to serum free media for overnight adaptation. Subsequently culture medium was changed to dexamethasone free medium 4h prior to stimulation. For stimulation, either TNF-α (R&D Systems) was added to a final concentration of 2 ng/ml, IL-1β (Jena Bioscience) at 20 ng/ml, and IL-17 (Novitec) at 100 ng/ml. When co-stimulation was performed, IL-17 was added to the culture medium 30 min before TNF-α was added. Accordingly, IL-17 alone was added to the cells for 1.5 or 4.5h, respectively. Three replicates of each experiment were performed.

Table S1: Experimental design of the microarray analysis[a]

Stimulation	IL-17	TNF-α	TNF-α/IL-17	IL-1ß	Control (untreated)
Concentration	100 ng/ml	2 ng/ml	IL-17: 100 ng/ml<br>TNF: 2ng/ml	20 ng/ml	-
Prestimulation	IL-17 30 min[b]	-	IL-17 30 min	-	
**Mouse 1+2**					
T1=1h	●	●	●	●	●
T2=4h	●	●	●	●	●
**Mouse 3+4**					
T1=1h	●	●	●	●	●
T2=4h	●	●	●	●	●
**Mouse 5+6**					
T1=1h	●	●	●	●	●
T2=4h	●	●	●	●	●

[a] Each point (●) represents one microarray derived from one RNA-preparation. For each treatment, timepoint and hepatocyte preparation a matching control was performed, which was used for point wise devision. Data from cells which were only treated with Il-17 were related to the 1 or 4 h control.
[b] that means, altogether these cells were treated with IL-17 either for 1.5 or 4.5 h

RNA preparation:

RNA was prepared from cultured hepatocytes with the Qiagen RNEasy plus Kit according to the manufactures instructions. For each sample, 2 million cells were used for RNA isolation and purification. Samples for microarray analysis were subjected to a quality control using an Agilent Bioanalyzer with the Pico RNA-Kit. Only RNA with a RNA integrity number (RIN) better than 8.2 was used for labeling and subsequent hybridization.

qReal-time PCR:

RNA isolation was performed as described above. 1 µg of total RNA was converted to single strand cDNA using Quantiscript Reverse Transcriptase (Qiagen) resulting in 100 µl diluted cDNA. The analysis of mRNA expression profiles was performed with multiplex quantitative real time PCR. In a 25 µl PCR reaction, 2 µl of cDNA (corresponding to 20 ng of total RNA input) was amplified in an Light Cycler 480 (Roche), using 2-fold QuantiTect Multiplex PCR Master Mix (Qiagen), 50 nM primers and 100 nM probe for the 18S rRNA reference gene (fwd: 5'-CGGCTACCACATCCAAGG-3', rev: 5'-CGGGTCGGGAGTGGGT, probe: 5'-TTGCGCGCCTGCTGCCT), and 300 nM primers and 100 nM probe for the gene of interest. The following target gene primers and probes were used (all from Sigma): mouse IκB-ξ (fwd: 5'-CAGTTGCCTGTCTTTCGTGA-3', rev: 5'-TGAATGGACTTCCCCTTCAG-3', probe: 5'-TATCGGGTGACACAGTTGGA-3') and mouse Cxcl2 (fwd: 5`-CCCCTGGTTCAGAAAATCATCC-3', rev: 5'-CTCTTTGGTTCTTCCGTTGAGG-3', probe: 5'-CCCAGGCTCCTCCTTTCCAGGTCA-3'). Semiquantitative PCR results were obtained using the delta cycle threshold (ΔCT) method and therefore reflect changes relative to untreated cells.

Preparation of the NF-κB lists

NF-κB target genes were identified using two different approaches: In the first approach, the publicly available gene collections of the Gilmore-Lab (http://www.nf-kb.org/) and the Touzet-Lab (http://bioinfo.lifl.fr/NF-KB/) were summarized. The resulting gene list was extended by target genes derived from scientific literature mining and analysis of experimental screening papers. Altogether 1033 NF-κB target genes were obtained (gene list on request by the authors).

The second approach was an *in silico* analysis based on the TFBS predictions as described in section Promoter analysis. Genes with promoter regions containing conserved TFBS predictions from NF-κB PWMs (MA0061, V$NFKAPPAB50_01, V$NFKAPPAB65_01,

V$NFKAPPAB_01, V$NFKB_C, V$NFKB_Q6) were classified as NF-κB target genes. Here, the list contained 3181 NF-κB target genes. However, filtering for phylogenetic conservation has the disadvantage that several genes were not annotated as NF-κB target genes although they are biochemically proven as NF-κB target genes. This may be due to low conserved NF-κB binding sites or rearrangements of promoter sections between the species or location of binding sites within introns. Differences in promoter architecture as shown for the Nfkbiz promoter, will also prevent annotation for those genes.

When comparing both approaches based on gene symbols, only a relatively small intersection was observed.

Reference

1. Klingmuller U, Bauer A, Bohl S, Nickel PJ, Breitkopf K, Dooley S, Zellmer S, Kern C, Merfort I, Sparna T, Donauer J, Walz G, Geyer M, Kreutz C, Hermes M, Gotschel F, Hecht A, Walter D, Egger L, Neubert K, Borner C, Brulport M, Schormann W, Sauer C, Baumann F, Preiss R, MacNelly S, Godoy P, Wiercinska E, Ciuclan L, Edelmann J, Zeilinger K, Heinrich M, Zanger UM, Gebhardt R, Maiwald T, Heinrich R, Timmer J, von WF, Hengstler JG: **Primary mouse hepatocytes for systems biology approaches: a standardized in vitro system for modelling of signal transduction pathways**. *Syst Biol (Stevenage)* 2006, **153:**433-447.
