# Supplementary material for: Genome-wide comparison between IL-17 and combined TNF-alpha/IL-17 induced genes in primary murine hepatocytes
Source: BMC Genomics. 2010 Apr 7;11:226. doi: 10.1186/1471-2164-11-226 (PMC2858152; doi:10.1186/1471-2164-11-226)
Supplement: Additional file 2 — Fold change values for all IL-17 significantly regulated genes. Table S2: Genes identified as upregulated and downregulated following IL-17 stimulation. [file 1471-2164-11-226-S2.PDF]

Additional File 2: Fold change values for all IL-17 significantly regulated genes

Table S2: Genes identified as upregulated and downregulated following IL-17 stimulation.

ANOVA IL17 upregulated genes (81) [transcripts: 92]

Affymetrix ID	Description	Gene Symbol	P-Value ANOVA	ARE-Cluster	IL1b 1h	TNF 1h	IL17 1h	TNF/IL17 1h	IL1b 4h	TNF 4h	IL17 4h	TNF/IL17 4h
1427348_at	zinc finger CCCH type containing 12A	Zc3h12a	2.78E-07		11.5	3.5	6.4	8.6	13.5	2.1	15.3	33.1
1417483_at	nuclear factor of kappa light polypeptide gene	*Nfkbiz	3.55E-05		43.6	4.5	11.0	27.5	4.1	1.3	8.3	9.8
1448162_at	vascular cell adhesion molecule 1	*Vcam1•	6.48E-05		21.1	9.5	2.2	8.0	9.9	13.5	1.7	7.5
1425829_a_at	STEAP family member 4	Steap4	9.59E-05		1.6	1.2	1.5	1.4	9.6	2.2	4.5	8.8
1460197_a_at	STEAP family member 4	Steap4	0.000106		1.8	1.4	1.5	1.4	10.5	2.5	5.4	10.8
1435133_at	UDP-glucose ceramide glucosyltransferase	Ugcg	0.0002		1.4	1.2	1.4	1.1	1.6	1.1	1.2	1.6
1420380_at	chemokine (C-C motif) ligand 2	*Ccl2•	0.000225		4.3	3.3	2.4	3.4	3.2	4.2	2.7	4.9
1441855_x_at	chemokine (C-X-C motif) ligand 1	*Cxcl1•	0.000276	2	7.4	4.0	3.5	6.0	2.4	1.9	2.6	4.1
1424101_at	heterogeneous nuclear ribonucleoprotein L	Hnrpl	0.000486		1.2	1.1	1.4	1.2	1.0	1.2	1.6	1.2
1426532_at	zinc finger, MYND domain containing 11	Zmynd11	0.000607	4	1.4	1.3	1.3	1.2	1.1	1.1	1.2	1.2
1423233_at	CCAAT/enhancer binding protein (C/EBP), del	Cebpd	0.000624		1.3	-1.9	1.5	1.4	4.2	-1.8	3.9	6.0
1423885_at	laminin, gamma 1	Lamc1•	0.000663		1.5	1.4	1.4	1.1	1.6	1.4	1.4	1.5
1450829_at	tumor necrosis factor, alpha-induced protein 3	*Tnfaip3	0.000671	5	12.9	7.5	1.8	7.2	2.3	3.0	2.3	4.9
1445457_at	Adult male cecum cDNA, RIKEN full-length en	1445457_at	0.000734		1.1	1.2	1.3	1.1	3.6	1.0	2.8	4.3
1457644_s_at	chemokine (C-X-C motif) ligand 1	*Cxcl1•	0.000814	2	7.5	4.7	4.0	6.5	3.2	2.3	3.4	4.9
1435777_at	RIKEN cDNA E030018N11 gene	E030018N11Rik•	0.000821		1.1	1.1	1.1	1.1	2.1	1.7	1.7	2.0
1420089_at	Nuclear factor of kappa light chain gene enhan	*Nfkbia•	0.001005	5	3.3	3.1	1.4	3.1	1.4	1.7	1.4	1.9
1419209_at	chemokine (C-X-C motif) ligand 1	*Cxcl1•	0.001076	2	7.3	4.2	3.6	6.2	2.7	2.0	3.1	4.8
1427081_at	RIKEN cDNA A630072M18 gene	A630072M18Rik	0.001146		1.4	1.2	1.2	1.0	1.7	1.5	1.6	1.9
1424384_a_at	zinc and ring finger 1	Znrf1•	0.001168		1.0	1.3	1.2	1.1	1.6	1.4	1.4	1.9
1427116_at	SET domain containing 1A	Setd1a	0.001216		-1.0	1.4	1.4	-1.0	1.0	1.2	1.4	-1.1
1415989_at	vascular cell adhesion molecule 1	*Vcam1•	0.001234	3	14.1	7.6	2.1	6.8	7.3	9.1	1.6	6.3
1448724_at	cytokine inducible SH2-containing protein	Cish	0.001356	3	3.7	2.6	1.7	2.6	1.0	1.3	1.4	1.3
1448239_at	heme oxygenase (decycling) 1	*Hmox1•	0.001503		2.8	2.1	1.7	2.0	2.0	2.4	1.7	2.1
1449119_at	ariadne homolog 2 (Drosophila)	Arih2	0.001538		1.1	1.0	1.1	1.2	-1.1	1.1	1.2	1.3
1426441_at	solute carrier family 11 (proton-coupled divaler	*Slc11a2	0.001759		1.4	1.1	1.0	1.1	3.1	1.5	2.1	3.1
1420723_at	vanin 3	Vnn3	0.001946		-1.0	1.0	-1.0	-1.0	4.1	1.0	1.9	4.6
1422027_a_at	E26 avian leukemia oncogene 1, 5' domain	Ets1•	0.002182	5	1.6	1.2	1.3	1.2	1.7	1.9	1.5	1.8
1416701_at	Rho family GTPase 3	Rnd3•	0.002186		2.2	1.9	1.7	2.1	1.4	1.1	1.6	1.6
1421109_at	camello-like 2	Cml2	0.002362		1.3	1.3	1.4	1.3	2.4	2.0	1.6	2.1
1453760_at	mesoderm induction early response 1 homolog	Mier1•	0.002403		1.2	1.3	1.3	1.4	1.3	1.1	1.2	1.2
1451727_at	SLU7 splicing factor homolog (S. cerevisiae)	Slu7	0.002442		1.3	1.0	1.6	1.3	1.4	1.6	1.3	1.4
1427559_a_at	activating transcription factor 2	Atf2	0.002477	5	1.3	1.4	1.3	1.5	1.2	1.1	1.3	1.2
1448328_at	SH3-domain binding protein 2	Sh3bp2	0.002543	5	1.1	1.1	1.2	1.1	1.7	1.5	1.4	1.9
1421859_at	a disintegrin and metallopeptidase domain 17	Adam17	0.002604		1.4	1.4	1.3	1.3	2.0	1.6	1.3	1.9
1434630_at	ankyrin repeat domain 28	Ankrd28	0.002618		1.2	1.2	1.2	1.1	1.1	1.1	1.2	1.2
1420928_at	beta galactoside alpha 2,6 sialyltransferase 1	St6gal1•	0.002807		1.5	1.4	1.3	1.1	1.9	1.8	1.6	2.1
1421064_at	membrane protein, palmitoylated 5 (MAGUK p	Mpp5•	0.002852		1.4	1.2	1.2	1.2	1.2	1.0	1.2	1.1
1447602_x_at	sulfatase 2	Sulf2	0.002866		1.5	1.3	1.4	1.3	1.6	1.5	1.4	1.4
1440739_at	vascular endothelial growth factor C	*Vegfc•	0.002931		1.7	1.4	1.4	1.3	1.7	1.8	1.4	1.8
1452078_a_at	solute carrier family 11 (proton-coupled divaler	*Slc11a2	0.002939	5	1.3	1.2	1.2	1.2	2.2	1.4	1.7	2.1
1454799_at	RIKEN cDNA A230097K15 gene	A230097K15Rik	0.003127		1.7	1.2	1.2	1.2	1.2	1.4	1.3	1.4
1419067_a_at	RAB guanine nucleotide exchange factor (GEF	Rabgef1	0.003207		1.4	1.4	1.3	1.2	1.3	1.2	1.2	1.3
1436546_at	RIKEN cDNA 6330549D23 gene	6330549D23Rik	0.003314		1.4	1.5	1.3	1.2	2.6	1.7	1.3	2.0
1423760_at	CD44 antigen	*Cd44•	0.003364		1.5	1.4	1.2	1.2	3.3	1.9	1.6	2.3
1434144_s_at	RIKEN cDNA 2410187C16 gene	2410187C16Rik	0.003399		1.1	1.1	1.5	1.3	1.5	1.0	1.5	1.6
1429262_at	Ras association (RalGDS/AF-6) domain family	Rassf6	0.003596	5	1.0	1.0	1.2	1.1	1.4	1.2	1.2	1.3
1441971_at	Transcribed locus	1441971_at•	0.003613		1.8	1.7	1.5	1.5	1.4	1.5	1.3	1.2
1450053_at	Kinesin family member 2A	Kif2a	0.003676		1.3	1.2	1.2	1.1	1.7	1.5	1.4	1.4
1426725_s_at	E26 avian leukemia oncogene 1, 5' domain	Ets1•	0.003743	5	2.7	2.0	1.8	1.6	1.8	2.2	1.5	1.9
1417268_at	CD14 antigen	*Cd14	0.003756		1.3	1.3	1.2	1.3	3.7	1.4	1.6	3.2
1449984_at	chemokine (C-X-C motif) ligand 2	*Cxcl2•	0.003766	1	104.8	34.8	3.9	54.0	13.9	6.2	4.9	34.5
1455331_at	RIKEN cDNA 9430067K14 gene	9430067K14Rik	0.003827		1.3	1.1	1.1	1.1	1.5	1.4	1.4	1.6
1426569_a_at	fyn-related kinase	Frk	0.003872		1.6	1.5	1.5	1.2	3.1	1.5	1.9	3.3
1452163_at	E26 avian leukemia oncogene 1, 5' domain	Ets1•	0.004025	5	2.4	2.0	1.9	1.8	1.6	2.1	1.3	1.7
1448306_at	nuclear factor of kappa light chain gene enhan	*Nfkbia•	0.004153	5	5.9	4.8	1.7	4.6	1.6	2.7	1.5	2.9
1460231_at	interferon regulatory factor 5	Irf5	0.004222	5	1.7	1.3	1.4	1.4	1.7	2.2	1.3	1.6
1433699_at	tumor necrosis factor, alpha-induced protein 3	*Tnfaip3	0.004473	5	14.7	9.7	2.2	9.2	2.0	2.6	1.9	4.1
1425913_a_at	RIKEN cDNA 2810022L02 gene	2810022L02Rik	0.004491		1.6	1.4	1.4	1.4	1.2	1.5	1.2	1.2
1460447_at	pseudouridylate synthase 7 homolog (S. cerev	Pus7l	0.004571		1.4	1.3	1.3	1.2	1.4	1.9	1.7	1.6
1451793_at	kelch-like 24 (Drosophila)	Klhl24	0.004719	5	1.2	1.2	-1.0	1.2	1.6	1.2	1.5	1.6
1419642_at	purine rich element binding protein B	Purb	0.004833		1.3	1.3	1.2	1.0	1.5	1.4	1.4	1.3
1451775_s_at	interleukin 13 receptor, alpha 1	Il13ra1•	0.004876	5	1.3	1.4	1.3	1.4	1.9	1.4	1.2	1.4
1434228_at	protein phosphatase 2C, magnesium depende	Ppm2c•	0.005073	5	-1.1	-1.0	1.1	-1.0	1.6	1.2	1.3	1.3
1420927_at	beta galactoside alpha 2,6 sialyltransferase 1	St6gal1•	0.005188		1.7	1.4	1.3	1.3	2.0	1.9	1.8	2.3
1421252_a_at	myocyte enhancer factor 2A	Mef2a	0.0052	5	1.3	1.3	1.2	1.2	-1.0	1.1	1.2	-1.1
1428084_at	KRR1, small subunit (SSU) processome comp	Krr1	0.005221		1.1	1.0	1.1	1.1	1.5	1.2	1.2	1.3
1422528_a_at	zinc finger protein 36, C3H type-like 1	Zfp36l1•	0.005284	4	1.8	1.5	1.5	1.5	1.2	1.3	1.3	1.3
1449623_at	thioredoxin reductase 3	Txnrd3	0.005502		1.3	1.3	1.4	1.3	1.5	1.2	1.3	1.3
1429100_at	RIKEN cDNA 2010109K11 gene	2010109K11Rik	0.005887		1.3	1.3	1.2	1.2	1.9	1.8	1.4	1.6
1426711_at	transmembrane and coiled-coil domains 3	Tmco3	0.005992		1.1	1.2	1.3	-1.1	1.1	1.3	1.2	1.1
1448596_at	solute carrier family 6 (neurotransmitter transp	Slc6a8•	0.005994		1.4	1.4	1.2	1.2	1.7	1.4	1.4	1.2
1450716_at	a disintegrin-like and metallopeptidase (reproly	Adamts1•	0.006023	5	1.7	1.2	1.1	1.1	1.1	1.1	1.3	1.6
1437329_at	protein tyrosine phosphatase-like (proline inste	Ptplb•	0.006231		1.4	1.2	1.3	1.2	1.8	1.6	1.3	1.5
1419647_a_at	immediate early response 3	*Ier3•	0.00637	5	6.6	3.4	1.8	4.0	1.3	1.4	1.5	2.2
1421269_at	UDP-glucose ceramide glucosyltransferase	Ugcg	0.006597	5	1.2	1.2	1.2	1.1	1.5	1.2	1.3	1.3

1455181_at	RAS p21 protein activator 2	Rasa2	0.006844		1.3	1.3	1.4	1.4	1.6	1.4	1.2	1.3
1423557_at	interferon gamma receptor 2	Ifngr2	0.007042		1.5	1.3	1.2	1.4	2.0	2.3	1.5	1.9
1417736_at	structural maintenance of chromosomes 6	**Smc6**	0.007228		1.2	1.2	1.1	-1.0	1.2	1.2	1.2	1.1
1416700_at	Rho family GTPase 3	Rnd3•	0.007734		2.0	1.7	1.6	1.8	1.5	1.0	1.6	1.7
1419132_at	toll-like receptor 2	*Tlr2	0.007942	5	5.5	3.5	1.7	3.8	7.9	6.4	2.0	7.6
1419030_at	ERO1-like (S. cerevisiae)	Ero1l	0.00797	5	1.4	1.4	1.2	1.2	1.4	1.2	1.3	1.1
1440169_x_at	interferon (alpha and beta) receptor 2	***Ifnar2•**	0.008051		2.7	1.8	1.3	1.6	2.2	1.6	1.6	2.8
1418133_at	B-cell leukemia/lymphoma 3	***Bcl3**	0.008108		1.9	1.4	1.1	1.5	2.4	2.7	1.9	2.5
1455727_at	zinc finger (CCCH type), RNA binding motif an	**Zrsr2•**	0.008249		1.1	1.0	1.1	-1.0	1.3	1.2	1.2	1.1
1450417_a_at	ribosomal protein S20 /// similar to 40S ribosor	Rps20	0.008277		1.5	1.6	1.5	1.5	1.3	1.2	1.2	1.1
1457883_at	Adult male aorta and vein cDNA, RIKEN full-le	1457883_at	0.008737		1.5	1.1	1.3	1.3	2.2	1.4	1.6	1.3
1418911_s_at	acyl-CoA synthetase long-chain family membe	Acsl4	0.009187	4	1.5	1.4	1.5	1.4	1.5	1.6	1.4	1.5
1454818_at	glucocorticoid modulatory element binding prot	Gmeb2	0.009494		1.5	1.3	1.3	1.1	1.5	1.4	1.3	1.3
1420609_at	membrane-associated ring finger (C3HC4) 7	**March7**	0.009645		1.2	1.2	1.2	1.2	1.1	1.0	1.0	-1.0
1438855_x_at	tumor necrosis factor, alpha-induced protein 2	***Tnfaip2•**	0.009691		10.3	4.2	1.4	4.7	5.7	3.5	2.0	7.2
1418718_at	chemokine (C-X-C motif) ligand 16	**Cxcl16•**	0.009913		1.6	1.3	1.4	1.4	2.6	2.6	1.8	2.8

Total: 24

bold SD>0.4

ARE containing genes (by ARES integrated DB)

Coloring code according to fold induction: 2.0 to 5.0; > 5.1; < -2.0

ANOVA IL17 downregulated genes (117) [transcripts: 127]

Affymetrix ID	Description	Gene Symbol	P-Value ANOVA	ARE-Cluster	IL1b 1h	TNF 1h	IL17 1h	TNF/IL17 1h	IL1b 4h	TNF 4h	IL17 4h	TNF/IL17 4h
1421097_at	endonuclease G	Endog	3.96E-07		-1.3	-1.3	-1.5	-1.2	-1.3	-1.2	-1.2	-1.2
1417398_at	related RAS viral (r-ras) oncogene homolog 2	Rras2	1.46E-05		-1.1	-1.1	-1.1	-1.2	-1.3	-1.2	-1.2	-1.3
1428071_at	RIKEN cDNA 1110038D17 gene	1110038D17Rik	4.82E-05		-1.5	-1.4	-1.4	-1.4	-1.6	-1.4	-1.3	-1.5
1438317_a_at	endonuclease G	Endog	9.33E-05		-1.3	-1.4	-1.4	-1.2	-1.3	-1.3	-1.3	-1.4
1448034_at	expressed sequence AI842396	AI842396	0.000108		-1.2	-1.3	-1.2	-1.2	-1.2	-1.1	-1.2	-1.2
1449966_s_at	calcium binding protein 39-like	Cab39l	0.000129		-1.4	-1.4	-1.3	-1.3	-1.6	-1.5	-1.5	-1.6
1425150_at	RIKEN cDNA C730036D15 gene	**C730036D15Rik**	0.000136		-1.7	-1.8	-1.5	-1.7	-2.9	-2.3	-1.9	-2.9
1427480_at	liver-expressed antimicrobial peptide 2	Leap2	0.000148		-2.0	-1.9	-1.9	-1.5	-1.9	-2.1	-2.4	-2.2
1428348_at	GDNF-inducible zinc finger protein 1	Gzf1	0.000156		-1.4	-1.3	-1.2	-1.2	-1.2	-1.2	-1.3	-1.4
1425281_a_at	TSC22 domain family 3	Tsc22d3•	0.000159		-1.6	-1.6	-1.4	-1.8	-1.8	-1.9	-1.8	-2.0
1445787_at	RIKEN cDNA 5033413D22 gene	5033413D22Rik	0.00017		-1.4	-1.6	-1.4	-1.2	-1.3	-1.5	-1.5	-1.7
1433700_at	RIKEN cDNA 4933433P14 gene	4933433P14Rik	0.000223		-1.3	-1.2	-1.3	-1.2	-1.4	-1.2	-1.3	-1.4
1448702_at	immediate early response 3 interacting protein	Ier3ip1	0.000234		-1.2	-1.1	-1.2	-1.1	-1.1	-1.1	-1.2	-1.1
1417761_at	apolipoprotein A-IV	Apoa4•	0.000241		-1.3	-1.6	-1.4	-1.5	-1.5	-1.3	-1.4	-1.3
1449062_at	ketohexokinase	Khk	0.000287		-1.4	-1.5	-1.4	-1.4	-1.3	-1.6	-1.5	-1.4
1419170_at	transmembrane protein 157	Tmem157	0.000383		-1.4	-1.4	-1.5	-1.2	-1.2	-1.2	-1.2	-1.3
1421022_x_at	acylphosphatase 1, erythrocyte (common) type	Acyp1	0.000386		-1.3	-1.3	-1.5	-1.3	-1.5	-1.5	-1.4	-1.4
1424953_at	cDNA sequence BC021614	BC021614	0.000386		-1.2	-1.3	-1.2	-1.3	-1.4	-1.1	-1.2	-1.1
1420772_a_at	TSC22 domain family 3	Tsc22d3•	0.000389		-1.4	-1.6	-1.3	-1.5	-1.8	-1.9	-1.6	-2.2
1453023_at	ankyrin repeat and KH domain containing 1	Ankhd1	0.000392	5	-1.5	-1.8	-1.7	-1.7	-2.0	-2.1	-2.1	-2.0
1417177_at	galactokinase 1	Galk1	0.000428		-1.4	-1.5	-1.5	-1.4	-1.3	-1.3	-1.2	-1.2
1416555_at	etoposide induced 2.4 mRNA	Ei24	0.000453		-1.1	-1.2	-1.3	-1.2	-1.2	-1.2	-1.1	-1.2
1435403_at	RIKEN cDNA 1700007L15 gene	1700007L15Rik	0.000469		-1.3	-1.5	-1.3	-1.2	-1.4	-1.4	-1.1	-1.3
1416953_at	connective tissue growth factor	***Ctgf•**	0.000498	5	-1.2	-1.3	-1.2	-1.3	-2.3	-1.6	-1.8	-2.3
1418138_at	sulfotransferase family 1D, member 1	Sult1d1	0.000635		-1.2	-1.4	-1.4	-1.1	-1.9	-1.6	-1.6	-1.8
1455869_at	Calcium/calmodulin-dependent protein kinase	Camk2b	0.000643		-1.3	-1.3	-1.2	-1.4	-1.3	-1.2	-1.3	-1.4
1449256_a_at	RAB11a, member RAS oncogene family	Rab11a•	0.000684	5	-1.2	-1.3	-1.5	-1.2	-1.0	-1.0	-1.0	-1.0
1451625_a_at	complement component 8, gamma subunit	C8g	0.000693		-1.2	-1.3	-1.3	-1.2	-1.0	-1.2	-1.3	-1.2
1429259_a_at	RIKEN cDNA 1810014B01 gene	1810014B01Rik•	0.000733		-1.2	-1.2	-1.3	-1.2	-1.2	-1.2	-1.2	-1.2
1448830_at	dual specificity phosphatase 1	Dusp1	0.000754	4	-2.2	-1.8	-1.5	-1.8	-1.3	-1.4	-1.4	-1.8
1440921_at	NACHT, LRR and PYD containing protein 12	**Nalp12**	0.000789		-2.3	-2.5	-1.8	-2.0	-5.5	-5.2	-3.0	-3.7
1438659_x_at	coiled-coil-helix-coiled-coil-helix domain contai	Chchd6	0.000944		-1.2	-1.1	-1.2	-1.0	-1.2	-1.2	-1.3	-1.2
1454836_at	transmembrane protein 18	Tmem18	0.001045		-1.3	-1.3	-1.3	-1.2	-1.2	-1.2	-1.4	-1.2
1419161_a_at	NADPH oxidase 4	Nox4	0.001048		-1.2	-1.2	-1.2	-1.2	-1.9	-1.5	-1.5	-1.7
1436504_x_at	apolipoprotein A-IV	Apoa4•	0.001071		-1.2	-1.4	-1.3	-1.3	-1.4	-1.3	-1.3	-1.2
1417937_at	dapper homolog 1, antagonist of beta-catenin (	Dact1•	0.001237		-1.0	-1.5	-1.4	-1.3	-1.3	-1.2	-1.2	-1.6
1448033_at	TatD DNase domain containing 1	Tatdn1	0.001245		-1.1	-1.2	-1.3	-1.0	-1.2	-1.1	-1.3	-1.1
1435874_at	gb:AV307561 /DB_XREF=gi:16393945 /DB_X	1435874_at	0.001424		-1.6	-1.4	-1.4	-1.2	-1.3	-1.1	-1.3	-1.3
1418992_at	coagulation factor X	F10	0.001566		-1.2	-1.3	-1.5	-1.2	-1.2	-1.1	-1.1	-1.2
1420124_s_at	T-cell leukemia translocation altered gene	Tcta	0.00157		-2.2	-1.8	-1.6	-1.7	-1.6	-1.4	-1.4	-1.4
1428263_a_at	transcription elongation factor B (SIII), polypep	Tceb2	0.00159		-1.1	-1.1	-1.2	-1.2	-1.1	-1.0	-1.1	-1.1
1418712_at	CDC42 effector protein (Rho GTPase binding)	Cdc42ep5	0.001673		-1.4	-1.3	-1.3	-1.2	-1.1	-1.2	-1.2	-1.2
1428023_at	RIKEN cDNA 3110009E18 gene	**3110009E18Rik**	0.001698		-1.1	-1.2	-1.5	-1.4	1.6	1.3	-1.2	1.1
1448973_at	sulfotransferase family 1D, member 1	Sult1d1	0.001708		-1.2	-1.3	-1.3	-1.2	-1.8	-1.7	-1.6	-1.9
1416765_s_at	mitochondria-associated protein involved in gr	Magmas	0.001977		-1.2	-1.3	-1.4	-1.2	-1.2	-1.1	-1.2	-1.1
1451385_at	RIKEN cDNA 2310056P07 gene	2310056P07Rik	0.00198		-1.3	-1.2	-1.3	-1.1	-1.1	-1.2	-1.2	-1.3
1422470_at	BCL2/adenovirus E1B interacting protein 1, NI	Bnip3	0.001995	5	-1.2	-1.2	-1.2	-1.3	-1.2	-1.5	-1.3	-1.5
1426452_a_at	RAB30, member RAS oncogene family	Rab30•	0.002427		-1.5	-1.2	-1.3	-1.1	-2.1	-1.5	-1.7	-2.2
1439352_at	tripartite motif protein 7	**Trim7**	0.002433		-1.4	-1.4	-1.2	-1.3	-2.5	-1.6	-1.3	-1.7
1435319_at	inositol hexaphosphate kinase 2	Ihpk2	0.002735	5	-1.7	-1.5	-1.5	-1.6	-1.3	-1.5	-1.2	-1.4
1451827_a_at	NADPH oxidase 4	Nox4	0.002938		-1.2	-1.2	-1.2	-1.2	-1.9	-1.6	-1.5	-1.8
1419905_s_at	hydroxyprostaglandin dehydrogenase 15 (NAD	**Hpgd**	0.003011		-1.3	-1.3	-1.3	-1.2	-3.3	-2.6	-1.8	-3.1
1451322_at	carboxymethylenebutenolidase-like (Pseudom	Cmbl	0.003128		-1.3	-1.2	-1.2	-1.1	-1.4	-1.4	-1.2	-1.4
1427213_at	6-phosphofructo-2-kinase/fructose-2,6-biphosp	Pfkfb1	0.003137		-1.4	-1.5	-1.3	-1.3	-1.9	-1.9	-1.5	-1.7
1457964_at	RIKEN cDNA 1810044D09 gene	1810044D09Rik•	0.003313		-1.3	-1.2	-1.4	-1.1	-1.4	-1.3	-1.6	-1.2
1436194_at	RIKEN cDNA C330008K14 gene	C330008K14Rik	0.003326		-1.4	-1.1	-1.3	-1.2	-1.2	-1.2	-1.1	-1.1
1424628_a_at	RIKEN cDNA 1500032D16 gene	1500032D16Rik	0.003348		-1.1	-1.2	-1.3	-1.1	-1.1	-1.1	-1.1	-1.1

1447936_at	RIKEN cDNA 2410006H16 gene	2410006H16Rik	0.003358		-1.7	-1.5	-1.4	-1.1	-1.4	-1.4	-1.4	-1.4
1428235_at	succinate dehydrogenase complex, subunit D,	Sdhd•	0.003485		-1.2	-1.2	-1.2	-1.1	-1.2	-1.2	-1.2	-1.3
1454921_at	gene model 561, (NCBI)	Gm561	0.003546		-1.4	-1.4	-1.5	-1.2	-1.2	-1.2	-1.3	-1.2
1417034_at	trafficking protein particle complex 6A	Trappc6a	0.003618		-1.4	-1.3	-1.5	-1.2	-1.3	-1.1	-1.1	-1.2
1460678_at	kelch domain containing 2	Klhdc2	0.003706		-1.2	-1.2	-1.3	-1.1	-1.3	-1.2	-1.2	-1.2
1448689_at	related RAS viral (r-ras) oncogene homolog 2	Rras2	0.003716		-1.1	-1.2	-1.2	-1.2	-1.5	-1.3	-1.3	-1.6
1451194_at	aldolase 2, B isoform	Aldob	0.00372		-1.5	-1.6	-1.4	-1.6	-1.3	-1.5	-1.4	-1.2
1434064_at	transmembrane protein 142C	Tmem142c	0.00373		-1.2	-1.2	-1.3	-1.2	-1.3	-1.1	-1.1	-1.1
1418918_at	insulin-like growth factor binding protein 1	*Igfbp1	0.003734		-1.2	-1.3	-1.4	-1.3	-1.9	-1.8	-1.4	-2.2
1449046_a_at	Josephin domain containing 2	Josd2•	0.003933		-1.0	-1.1	-1.4	-1.1	-1.2	-1.1	-1.2	-1.1
1435007_s_at	expressed sequence AI132487	AI132487	0.003997		-1.6	-1.5	-1.3	-1.4	-2.4	-1.9	-1.7	-2.2
1419549_at	arginase 1, liver	Arg1	0.004041	NA	-1.2	-1.4	-1.3	-1.3	-1.6	-1.6	-1.6	-1.7
1431192_at	RIKEN cDNA 2010203O03 gene	2010203O03Rik	0.00412		-1.3	-1.2	-1.1	-1.2	-1.6	-1.4	-1.4	-1.5
1457915_at	RIKEN cDNA 4833442J19 gene	4833442J19Rik	0.004137		-1.5	-1.5	-1.3	-1.3	-1.3	-1.6	-1.2	-1.4
1436949_a_at	transcription elongation factor B (SIII), polypep	Tceb2	0.004153		-1.2	-1.2	-1.3	-1.2	-1.1	-1.0	-1.1	-1.0
1452678_a_at	cysteine conjugate-beta lyase 1	Ccbl1	0.004304	1	-1.3	-1.3	-1.3	-1.2	-1.9	-2.1	-1.7	-2.2
1428874_at	RIKEN cDNA 1110019N10 gene	**1110019N10Rik•**	0.004353		-1.2	-1.2	-1.4	-1.1	1.1	1.0	-1.1	1.1
1424497_at	RIKEN cDNA 1110032O16 gene	1110032O16Rik	0.004483		-1.2	-1.4	-1.4	-1.2	-1.1	-1.1	-1.3	-1.2
1418701_at	catechol-O-methyltransferase	Comt•	0.004533		-1.3	-1.5	-1.5	-1.4	-1.4	-1.2	-1.1	-1.1
1452184_at	NADH dehydrogenase (ubiquinone) 1 beta sul	Ndufb9•	0.004595		-1.3	-1.4	-1.3	-1.2	-1.2	-1.1	-1.2	-1.1
1449108_at	ferredoxin 1	Fdx1	0.004635	4	-1.2	-1.3	-1.2	-1.3	-1.2	-1.2	-1.2	-1.2
1453269_at	unc-5 homolog B (C. elegans)	**Unc5b•**	0.004644		1.2	-1.0	-1.1	-1.0	-1.8	-1.3	-1.4	-1.6
1428163_at	SAR1 gene homolog B (S. cerevisiae)	Sar1b	0.004744		-1.3	-1.2	-1.3	-1.1	-1.3	-1.3	-1.2	-1.3
1419618_at	butyrobetaine (gamma), 2-oxoglutarate dioxyg	Bbox1	0.004862		-1.5	-1.8	-1.6	-1.5	-1.9	-1.7	-1.5	-1.5
1426367_at	calcium binding protein 39-like	Cab39l	0.005133		-1.2	-1.3	-1.2	-1.2	-1.5	-1.5	-1.5	-1.6
1428296_at	polymerase (RNA) II (DNA directed) polypeptic	**Polr2l**	0.005251		-1.1	-1.2	-1.2	-1.2	-1.0	1.0	-1.1	-1.0
1435477_s_at	Fc receptor, IgG, low affinity IIb	Fcgr2b	0.005435		-1.2	-1.2	-1.2	-1.2	-2.0	-1.5	-1.3	-1.3
1423833_a_at	brain protein 44	Brp44	0.005442		-1.3	-1.3	-1.3	-1.3	-1.2	-1.2	-1.2	-1.2
1428322_a_at	NADH dehydrogenase (ubiquinone) 1 beta sul	Ndufb10	0.005521		-1.2	-1.2	-1.3	-1.2	-1.1	-1.1	-1.1	-1.1
1449418_s_at	F-box protein 36	Fbxo36•	0.005575		-1.3	-1.2	-1.2	-1.2	-1.2	-1.3	-1.4	-1.4
1420123_at	T-cell leukemia translocation altered gene	Tcta	0.005654		-2.0	-1.6	-1.5	-1.4	-1.4	-1.1	-1.1	-1.2
1448985_at	dual specificity phosphatase 22	**Dusp22•**	0.005866		-1.0	-1.1	-1.2	1.0	-1.0	-1.1	-1.2	-1.1
1419127_at	neuropeptide Y	Npy	0.005901		-1.1	-1.1	-1.2	-1.3	-1.7	-1.5	-1.5	-1.4
1424164_at	mitochondrial ribosomal protein L50	Mrpl50	0.005994		-1.2	-1.2	-1.2	-1.2	-1.2	-1.3	-1.2	-1.2
1448609_at	thiosulfate sulfurtransferase, mitochondrial	Tst	0.006084		-1.1	-1.2	-1.2	-1.1	-1.3	-1.1	-1.1	-1.2
1435431_at	RIKEN cDNA 2310047M15 gene	2310047M15Rik	0.006335		-1.3	-1.4	-1.3	-1.2	-1.1	-1.1	-1.2	-1.1
1428465_at	transmembrane protein 147	Tmem147	0.006346		-1.2	-1.4	-1.4	-1.2	-1.3	-1.2	-1.2	-1.2
1425560_a_at	S100 calcium binding protein A16	S100a16	0.006359		-1.2	-1.3	-1.3	-1.2	-1.2	-1.2	-1.3	-1.2
1435524_at	RIKEN cDNA 2010109N14 gene	2010109N14Rik•	0.006714		-1.2	-1.2	-1.3	-1.0	-1.2	-1.2	-1.2	-1.1
1428214_at	translocase of outer mitochondrial membrane 7	**Tomm7**	0.006837		-1.2	-1.2	-1.3	-1.2	1.0	1.0	-1.1	-1.1
1428215_x_at	translocase of outer mitochondrial membrane 7	Tomm7	0.006956		-1.2	-1.2	-1.2	-1.1	-1.1	-1.0	-1.1	-1.1
1421092_at	serine (or cysteine) peptidase inhibitor, clade A	Serpina12	0.007143		-1.7	-1.5	-1.4	-1.4	-1.3	-1.3	-1.3	-1.2
1424303_at	DEP domain containing 7	Depdc7	0.007245		-1.2	-1.2	-1.3	-1.2	-1.3	-1.4	-1.2	-1.3
1428360_x_at	NADH dehydrogenase (ubiquinone) 1 alpha su	Ndufa7	0.007358		-1.3	-1.3	-1.3	-1.2	-1.1	-1.1	-1.1	-1.1
1428228_at	phosphoglucomutase 3	Pgm3•	0.007482	5	-1.1	-1.3	-1.2	-1.2	-1.5	-1.3	-1.3	-1.4
1448961_at	phospholipid scramblase 2	**Plscr2**	0.007529		-1.2	-1.3	-1.4	-1.1	-3.2	-2.1	-1.8	-2.5
1452426_x_at	Zinc finger protein 236 /// CDNA clone IMAGE:	**Zfp236**	0.007563		-1.0	-1.1	-1.2	-1.0	1.3	1.2	-1.2	1.1
1428710_at	Ras-like without CAAX 1	**Rit1**	0.007567	5	-1.6	-1.5	-1.5	-1.4	1.0	-1.5	-1.0	-1.1
1422178_a_at	RAB17, member RAS oncogene family	Rab17	0.007635		-1.4	-1.4	-1.4	-1.3	-1.4	-1.5	-1.2	-1.4
1419994_s_at	DNA segment, Chr 10, ERATO Doi 641, expre	D10Ertd641e	0.007812		-1.2	-1.1	-1.1	-1.1	-1.2	-1.0	-1.1	-1.1
1425455_a_at	churchill domain containing 1	Churc1	0.007864		-1.3	-1.3	-1.4	-1.2	-1.2	-1.2	-1.3	-1.2
1452599_s_at	expressed sequence AI413582	AI413582	0.007923		-1.5	-1.4	-1.5	-1.3	-1.2	-1.0	-1.2	-1.3
1424189_at	phosphatidylinositol glycan anchor biosynthesi	Pigc	0.007967		-1.4	-1.3	-1.4	-1.2	-1.0	-1.1	-1.1	-1.1
1424354_at	transmembrane protein 140	**Tmem140**	0.008114		-2.3	-1.6	-1.6	-1.7	1.1	1.0	-1.2	-1.4
1428309_s_at	p53 and DNA damage regulated 1	Pdrg1•	0.008375		-2.1	-1.6	-1.4	-1.4	-1.1	-1.2	-1.2	-1.1
1426567_a_at	PQ loop repeat containing 1	Pqlc1	0.008407		-1.1	-1.1	-1.1	-1.1	-1.5	-1.3	-1.2	-1.5
1425591_a_at	chromatin modifying protein 2A	Chmp2a	0.008499		-1.2	-1.2	-1.3	-1.2	-1.1	-1.1	-1.2	-1.1
1452585_at	mitochondrial ribosomal protein S28	Mrps28•	0.008602		-1.2	-1.2	-1.2	-1.1	-1.1	-1.0	-1.1	-1.0
1417042_at	solute carrier family 37 (glycerol-6-phosphate t	Slc37a4	0.008868		-1.3	-1.4	-1.3	-1.3	-1.6	-1.7	-1.7	-1.7
1429568_x_at	ubiquitin-conjugating enzyme E2F (putative)	**Ube2f**	0.008984		1.1	1.0	-1.1	1.1	1.0	1.0	-1.1	1.0
1417064_at	jagunal homolog 1 (Drosophila)	Jagn1	0.009		-1.2	-1.2	-1.2	-1.2	-1.2	-1.1	-1.2	-1.1
1425201_a_at	hydroxypyruvate isomerase homolog (E. coli)	Hyi	0.009088		-1.3	-1.3	-1.4	-1.1	-1.1	-1.1	-1.1	-1.1
1440587_at	gb:BM238838 /DB_XREF=gi:17874108 /DB_X	**1440587_at**	0.009186		-2.0	-2.0	-1.5	-1.8	-2.6	-1.1	-2.0	-1.6
1456204_at	RIKEN cDNA 2010107H07 gene	2010107H07Rik	0.00933		-1.3	-1.4	-1.5	-1.2	-1.5	-1.4	-1.3	-1.3
1449048_s_at	RAB4A, member RAS oncogene family	Rab4a	0.009421		-1.1	-1.3	-1.2	-1.2	-1.3	-1.2	-1.2	-1.3
1448643_at	Sjogren's syndrome nuclear autoantigen 1	Ssna1	0.009464		-1.3	-1.3	-1.2	-1.3	-1.1	-1.1	-1.1	-1.2
1421498_a_at	RIKEN cDNA 2010204K13 gene	2010204K13Rik	0.009536		-1.1	-1.0	-1.2	-1.0	-1.3	-1.1	-1.2	-1.1
1439496_at	RIKEN cDNA 4921524J06 gene	4921524J06Rik	0.009792		-1.0	-1.2	-1.3	-1.3	-1.4	-1.4	-1.4	-1.4
1449338_at	DNA segment, Chr 10, ERATO Doi 641, expre	D10Ertd641e	0.009881		-1.4	-1.3	-1.3	-1.2	-1.3	-1.2	-1.2	-1.2
1417219_s_at	thymosin, beta 10	Tmsb10•	0.009906		-1.3	-1.2	-1.2	-1.1	-1.4	-1.3	-1.3	-1.3
				Total: 11								

bold SD>0.4

Color code fold induction: -1.5 - -3; > -3.1

Analysis of NF-kappaB target genes

		NF-kappaB target genes		
	total	all	Lit-List	Bioinf.
upregulated	92	44 (48%)	21 (23%)	36 (39%)
downregulated	127	23 (18%)	2 (2%)	23 (18%)

For better comparison, fold induction ratios resulting from the other stimuli are also presented. The color code used to visualize expression values is indicated below each table. The number of identified NF-κB target genes are separately summarized for IL-17 up- and downregulated genes for both NF-κB target gene lists. Percentages relative to the corresponding list for all NF-κB target genes are indicated in brackets. Different time points are not considered by the ANOVA.
