# Supplementary material for: Genome-wide comparison between IL-17 and combined TNF-alpha/IL-17 induced genes in primary murine hepatocytes
Source: BMC Genomics. 2010 Apr 7;11:226. doi: 10.1186/1471-2164-11-226 (PMC2858152; doi:10.1186/1471-2164-11-226)
Supplement: Additional file 3 — Genes up-regulated by combined TNF-α and IL-17 stimulation. Table S3: Genes up-regulated by combined TNF-α and IL-17 stimulation. [file 1471-2164-11-226-S3.PDF]

Additional file 3: Genes up-regulated by combined TNF-α and IL-17 stimulation.

Table S3. Genes up-regulated by combined TNF-α and IL-17 stimulation

Name	Gene Symbol	Description	IL1b 1h	TNF 1h	IL17 1h	TNF+IL17 1h	IL1b 4h	TNF 4h	IL17 4h	TNF+IL17 4h
1449984_at	*Cxcl2•	chemokine (C-X-C motif) ligand 2	104,8	34,8	3,9	54,0	13,9	6,2	4,9	34,5
1427348_at	Zc3h12a	zinc finger CCCH type containing 12A	11,5	3,5	6,4	8,6	13,5	2,1	15,3	33,1
1460197_a_at	Steap4	STEAP family member 4	1,8	1,4	1,5	1,4	10,5	2,5	5,4	10,8
1417483_at	*Nfkbiz	IkappaB zeta	43,6	4,5	11,0	27,5	4,1	1,3	8,3	9,8
1425829_a_at	Steap4	STEAP family member 4	1,6	1,2	1,5	1,4	9,6	2,2	4,5	8,8
1419132_at	*Tlr2	toll-like receptor 2	5,5	3,5	1,7	3,8	7,9	6,4	2,0	7,6
1448162_at	*Vcam1•	vascular cell adhesion molecule 1	21,1	9,5	2,2	8,0	9,9	13,5	1,7	7,5
1438855_x_at	*Tnfaip2•	tumor necrosis factor, alpha-induced protein 2	10,3	4,2	1,4	4,7	5,7	3,5	2,0	7,2
1415989_at	*Vcam1•	vascular cell adhesion molecule 1	14,1	7,6	2,1	6,8	7,3	9,1	1,6	6,3
1423233_at	Cebpd	CCAAT/enhancer binding protein (C/EBP), delta	1,3	-1,9	1,5	1,4	4,2	-1,8	3,9	6,0
1423017_a_at	*Il1rn•	interleukin 1 receptor antagonist	2,3	1,4	1,3	1,2	6,3	1,9	1,8	5,0
1420380_at	*Ccl2•	chemokine (C-C motif) ligand 2	4,3	3,3	2,4	3,4	3,2	4,2	2,7	4,9
1450829_at	*Tnfaip3	tumor necrosis factor, alpha-induced protein 3	12,9	7,5	1,8	7,2	2,3	3,0	2,3	4,9
1457644_s_at	*Cxcl1•	chemokine (C-X-C motif) ligand 1	7,5	4,7	4,0	6,5	3,2	2,3	3,4	4,9
1419209_at	*Cxcl1•	chemokine (C-X-C motif) ligand 1	7,3	4,2	3,6	6,2	2,7	2,0	3,1	4,8
1420723_at	Vnn3	vanin 3	-1,0	1,0	-1,0	-1,0	4,1	1,0	1,9	4,6
1445457_at	1445457_at	clone 9130219A07 hypothetical protein	1,1	1,2	1,3	1,1	3,6	1,0	2,8	4,3
1441855_x_at	*Cxcl1•	chemokine (C-X-C motif) ligand 1	7,4	4,0	3,5	6,0	2,4	1,9	2,6	4,1
1433699_at	*Tnfaip3	tumor necrosis factor, alpha-induced protein 3	14,7	9,7	2,2	9,2	2,0	2,6	1,9	4,1
1419196_at	Hamp1•	hepcidin antimicrobial peptide 1	-1,1	-1,2	-1,5	-1,0	6,4	-1,1	1,8	4,1
1427705_a_at	*Nfkb1	nuclear factor of kappa light chain gene enhancer in B-cells 1, p105	1,7	1,2	1,1	1,3	3,8	3,0	1,8	3,8
1421362_a_at	Frk	fyn-related kinase	1,8	1,4	1,4	1,5	3,3	1,3	1,6	3,6
1424067_at	*Icam1•	intercellular adhesion molecule	6,2	4,2	1,9	4,3	3,4	3,8	1,3	3,4
1450173_at	*Ripk2	receptor (TNFRSF)-interacting serine-threonine kinase 2	4,9	2,8	1,3	3,1	3,7	2,1	1,4	3,3

1426570_a_at	Frk	fyn-related kinase	1,6	1,3	1,2	1,1	3,8	1,5	1,9	3,3
1426569_a_at	Frk	fyn-related kinase	1,6	1,5	1,5	1,2	3,1	1,5	1,9	3,3
1455197_at	Rnd1•	Rho family GTPase 1	8,6	4,1	1,9	5,2	2,3	2,5	1,2	3,2
1435040_at	Irak3	interleukin-1 receptor-associated kinase 3	1,5	1,3	1,2	1,2	4,1	2,2	1,7	3,2
1417268_at	*Cd14	CD14 antigen	1,3	1,3	1,2	1,3	3,7	1,4	1,6	3,2
1458299_s_at	*Nfkbie	nuclear factor of kappa light polypeptide gene enhancer in B-cells inhibitor, epsilon	4,1	2,8	1,4	2,9	3,5	6,1	1,3	3,1
1426441_at	*Slc11a2	solute carrier family 11 (proton-coupled divalent metal ion transporters), member 2	1,4	1,1	1,0	1,1	3,1	1,5	2,1	3,1
1419197_x_at	Hamp1•	hepcidin antimicrobial peptide 1	-1,2	-1,1	-1,2	1,0	4,6	-1,1	1,4	3,0
1418099_at	*Tnfrsf1b	tumor necrosis factor receptor superfamily, member 1b	1,4	1,1	1,2	1,2	3,0	1,4	1,8	3,0
1419319_at	Saa4	serum amyloid A 4	1,1	1,1	1,1	1,0	3,2	1,1	1,4	3,0
1436413_at	Frk	fyn-related kinase	1,6	1,7	1,3	1,1	2,9	1,2	1,4	2,9
1448306_at	*Nfkbia•	nuclear factor of kappa light chain gene enhancer in B-cells inhibitor, alpha	5,9	4,8	1,7	4,6	1,6	2,7	1,5	2,9
1422677_at	Dgat2	diacylglycerol O-acyltransferase 2	1,3	1,1	-1,0	1,1	3,1	1,3	1,2	2,8
1449731_s_at	*Nfkbia•	nuclear factor of kappa light chain gene enhancer in B-cells inhibitor, alpha	5,1	4,0	1,4	4,2	1,5	2,6	1,4	2,8
1418718_at	Cxcl16•	chemokine (C-X-C motif) ligand 16	1,6	1,3	1,4	1,4	2,6	2,6	1,8	2,8
1434376_at	*Cd44•	CD44 antigen	1,3	1,3	1,0	1,0	3,1	1,8	1,4	2,8
1440169_x_at	*Ifnar2•	interferon (alpha and beta) receptor 2	2,7	1,8	1,3	1,6	2,2	1,6	1,6	2,8
1438676_at	Mpa2l	macrophage activation 2 like /// similar to macrophage activation 2 like /// similar to macrophage activation 2 like	1,8	1,5	1,4	1,6	5,0	2,4	1,2	2,7
1418674_at	*Osmr	oncostatin M receptor	1,9	1,6	1,6	1,5	3,3	2,4	1,5	2,7
1422678_at	Dgat2	diacylglycerol O-acyltransferase 2	1,4	1,1	1,0	1,1	2,9	1,3	1,2	2,7
1416630_at	Id3•	inhibitor of DNA binding 3	4,0	2,7	1,5	3,5	2,0	1,7	1,7	2,6
1449195_s_at	Cxcl16•	chemokine (C-X-C motif) ligand 16	1,4	1,2	1,1	1,3	2,2	2,2	1,5	2,5
1438157_s_at	*Nfkbia•	nuclear factor of kappa light chain gene enhancer in B-cells inhibitor, alpha	4,0	3,4	1,7	3,2	1,5	2,4	1,4	2,5
1448560_at	Bid	BH3 interacting domain death agonist	1,3	1,1	1,1	1,2	2,8	2,2	1,2	2,5
1454254_s_at	1600029D21Rik	RIKEN cDNA 1600029D21 gene	1,5	1,1	1,1	1,2	2,5	1,6	1,9	2,5
1418133_at	*Bcl3	B-cell leukemia/lymphoma 3	1,9	1,4	1,1	1,5	2,4	2,7	1,9	2,5
1450826_a_at	*Saa3	serum amyloid A 3	1,1	-1,1	-1,2	-1,0	3,2	1,1	-1,1	2,4

1450767_at	Nedd9	neural precursor cell expressed, developmentally down-regulated gene 9	1,5	1,7	1,2	1,3	1,9	3,8	2,1	2,4
1417045_at	Bid	BH3 interacting domain death agonist	1,2	1,2	1,1	1,2	2,9	2,3	1,2	2,4
1455899_x_at	*Socs3	suppressor of cytokine signaling 3	5,5	1,6	1,2	2,5	2,5	1,5	1,9	2,3
1423760_at	*Cd44•	CD44 antigen	1,5	1,4	1,2	1,2	3,3	1,9	1,6	2,3
1420927_at	St6gal1•	beta galactoside alpha 2,6 sialyltransferase 1	1,7	1,4	1,3	1,3	2,0	1,9	1,8	2,3
1450703_at	Slc7a2	solute carrier family 7 (cationic amino acid transporter, y+ system), member 2	1,6	1,3	-1,1	1,4	5,6	2,9	1,3	2,3

Upregulated 1.5 - 3 fold

upregulated >3fold

For comparison, fold factors for other stimulations are also presented. Primary hepatocytes were prestimulated with IL-17 (100ng/ml) for 30 min, followed by addition of TNF-α (2ng/ml) for another 1 or 4h. Genes up-regulated more than 2fold are listed. Fold factors are related to the time matched control. NF-κB target genes are indicated as (*) for literature derived annotation or as a dot (•) for bioinformatical annotation.
