# Supplementary material for: Genome-wide comparison between IL-17 and combined TNF-alpha/IL-17 induced genes in primary murine hepatocytes
Source: BMC Genomics. 2010 Apr 7;11:226. doi: 10.1186/1471-2164-11-226 (PMC2858152; doi:10.1186/1471-2164-11-226)
Supplement: Additional file 4 — Genes showing synergistic or inhibitory regulation following combined TNF-α/IL-17 treatment. Table S4: Genes showing synergistic/inhibitory regulation following combined TNF-α/IL-17 treatment. [file 1471-2164-11-226-S4.PDF]

Additional file 4: Genes showing synergistic or inhibitory regulation following combined TNF-α/IL-17 treatment

Table S4: Genes showing synergistic/inhibitory regulation following combined TNF-α/IL-17 treatment.

Affy-ID	Description	Gene Symbol	IL1 1h	SD	IL1 4h	SD	TNF 1h	SD	TNF 4h	SD	IL17 1h	SD	IL17 4h	SD	Tnf&IL17 1h	SD	Tnf&IL17 4h	SD
**Synergy 1h**																		
1417483_at	IkappaBzeta	*Nfkbiz	43.62	28.24	5.49	2.09	4.45	2.89	1.27	0.11	11.01	6.46	8.25	0.21	27.53	18.51	9.78	3.47
1449984_at	chemokine (C-X-C motif) ligand 2	*Cxcl2•	104.84	145.25	20.22	1.96	34.76	49.87	6.19	1.21	3.94	3.75	4.91	1.33	53.95	69.31	34.52	15.38
**Synergy 4h**																		
1425829_a_at	STEAP family member 4	Steap4	1.58	0.82	10.46	1.83	1.19	0.33	2.17	0.39	1.52	0.37	4.46	0.90	1.42	0.31	8.81	3.11
1427348_at	zinc finger CCCH type containing 12A	Zc3h12a	11.47	4.14	14.29	8.58	3.54	0.71	2.12	0.52	6.43	0.99	15.26	7.79	8.64	5.80	33.12	2.78
1438148_at	gene model 1960, (NCBI)	Gm1960	NA		17.80	39.80	NA		6.24	5.14	NA		8.11	2.68	NA		39.22	4.66
1449984_at	chemokine (C-X-C motif) ligand 2	*Cxcl2•	104.84	145.25	20.22	1.96	34.76	49.87	6.19	1.21	3.94	3.75	4.91	1.33	53.95	69.31	34.52	15.38
1460197_a_at	STEAP family member 4	Steap4	1.81	0.93	11.65	5.88	1.38	0.37	2.50	0.62	1.55	0.36	5.37	2.18	1.43	0.24	10.78	1.97
**Inhibitory 1h**																		
1415989_at	vascular cell adhesion molecule 1	*Vcam1•	14.09	8.98	5.89	2.45	7.56	4.76	9.07	0.70	2.12	0.67	1.56	0.49	6.80	2.82	6.29	1.40
1419575_s_at	zinc finger protein 292	Zfp292	1.26	0.37	1.04	0.05	1.56	0.32	0.88	0.04	1.64	0.42	0.92	0.34	1.19	0.64	0.87	0.22
1420380_at	chemokine (C-C motif) ligand 2	*Ccl2•	4.31	2.11	2.98	1.24	3.32	1.44	4.23	1.50	2.39	1.03	2.74	0.23	3.35	1.36	4.93	0.92
1426725_s_at	E26 avian leukemia oncogene 1, 5' domain	Ets1•	2.73	0.96	1.65	0.37	2.03	0.81	2.22	0.34	1.76	0.64	1.47	0.51	1.63	0.62	1.88	0.35
1428625_a_at	RMI1, RecQ mediated genome instability 1, homolog (S. cerevisiae)	Rmi1	0.99	0.80	NA	0.12	1.39	0.93	1.14	0.19	1.62	1.00	1.42	0.62	1.00	0.62	1.46	0.18
1433699_at	tumor necrosis factor, alpha-induced protein 3	*Tnfaip3	14.73	7.52	2.74	0.23	9.67	2.94	2.58	0.53	2.18	0.18	1.90	0.31	9.22	2.73	4.13	0.82
1435180_at	podocan	Podn	1.33	0.59	NA		1.52	0.68	1.23	0.85	1.75	0.84	1.30	0.73	1.22	0.29	0.99	0.48
1435906_x_at	guanylate nucleotide binding protein 2	*Gbp2	2.08	1.05	2.29	0.58	1.99	1.02	2.35	0.48	1.62	0.63	1.39	0.80	1.51	0.59	1.78	0.64
1440984_at	bromodomain adjacent to zinc finger domain, 2B	Baz2b	1.68	0.05	1.15	0.53	1.74	0.21	1.25	0.46	1.72	0.55	1.10	0.67	1.32	0.09	1.14	0.24
1444980_at	one cut domain, family member 2	Onecut2•	1.80	0.86	1.12	0.46	1.99	0.74	1.42	0.03	1.41	0.20	1.05	0.80	1.27	0.51	1.04	0.23
1447643_x_at	snail homolog 2 (Drosophila)	Snai2•	1.63	2.18	1.33	1.49	2.00	0.99	1.17	0.89	1.73	1.07	0.82	0.34	1.50	0.41	0.69	0.57
1448162_at	vascular cell adhesion molecule 1	*Vcam1•	21.05	11.97	7.28	3.66	9.53	8.00	13.48	1.62	2.25	0.68	1.69	0.33	8.03	6.45	7.51	3.30
1450829_at	tumor necrosis factor, alpha-induced protein 3	*Tnfaip3	12.92	5.33	2.99	0.22	7.54	1.28	2.97	0.46	1.78	0.30	2.31	0.65	7.20	1.76	4.88	0.81
1451048_at	methionine aminopeptidase 2	Metap2•	1.46	0.69	1.43	0.22	1.72	0.36	1.34	0.33	1.70	0.84	1.37	0.43	1.34	0.61	1.47	0.09
1452163_at	E26 avian leukemia oncogene 1, 5' domain	Ets1•	2.37	0.72	1.56	0.23	2.00	0.63	2.15	0.16	1.85	0.71	1.34	0.43	1.81	0.86	1.69	0.26
1457644_s_at	chemokine (C-X-C motif) ligand 1	*Cxcl1•	7.49	6.03	3.87	2.36	4.72	3.16	2.33	0.84	4.04	3.73	3.41	1.26	6.53	4.97	4.88	2.55
**Inhibitory 4h**																		
1415989_at	vascular cell adhesion molecule 1	*Vcam1•	14.09	8.98	5.89	2.45	7.56	4.76	9.07	0.70	2.12	0.67	1.56	0.49	6.80	2.82	6.29	1.40
1418133_at	B-cell leukemia/lymphoma 3	*Bcl3	1.90	0.51	2.69	0.61	1.44	0.26	2.72	0.19	1.12	0.21	1.91	0.41	1.52	0.12	2.48	0.31
1418930_at	chemokine (C-X-C motif) ligand 10	*Cxcl10	10.33	7.31	0.62	0.35	4.75	2.18	4.11	1.27	1.81	0.23	0.94	0.19	5.74	2.37	1.13	0.93
1420380_at	chemokine (C-C motif) ligand 2	*Ccl2•	4.31	2.11	2.98	1.24	3.32	1.44	4.23	1.50	2.39	1.03	2.74	0.23	3.35	1.36	4.93	0.92
1425687_at	CASP8 and FADD-like apoptosis regulator	Cflar	1.11	0.29	1.65	0.76	1.01	0.20	1.78	0.57	NA		1.66	0.32	0.92	0.35	1.28	0.46
1427473_at	glutathione S-transferase, mu 3	Gstm3	1.60	1.13	1.68	0.98	1.20	0.74	1.88	0.37	1.19	0.37	1.91	0.52	1.29	0.59	1.53	0.52
1431843_a_at	nuclear factor of kappa light polypeptide gene enhancer in B-cells inhibitor, epsilon	*Nfkbie	2.99	1.81	2.37	0.67	2.03	1.15	4.74	0.20	1.06	0.39	1.20	0.19	2.35	0.49	1.94	0.15
1435213_at	NHL repeat containing 1	Nhlrc1•	1.11	0.19	2.02	1.37	1.05	0.45	2.12	0.99	1.05	0.29	1.70	1.11	1.00	0.38	1.63	1.24
1436030_at	cache domain containing 1	Cachd1	1.72	0.65	1.15	0.84	1.32	0.18	2.00	0.39	1.26	0.44	1.52	0.60	1.08	0.25	1.32	0.58
1436031_at	cache domain containing 1	Cachd1	1.66	0.37	1.33	1.02	1.29	0.21	2.16	0.51	1.31	0.25	1.57	0.70	1.12	0.12	1.50	0.73
1437132_x_at	neural precursor cell expressed, developmentally down-regulated gene 9	Nedd9	2.64	4.38	1.09	0.38	1.78	1.27	2.25	0.82	1.77	1.73	1.24	0.53	1.98	1.76	1.42	0.94
1439260_a_at	ectonucleotide pyrophosphatase/ phosphodiesterase 3	Enpp3	1.49	0.92	1.21	0.54	1.37	0.57	1.80	1.45	1.40	0.90	1.58	0.92	1.14	0.92	1.31	1.06
1441946_at	inter-alpha (globulin) inhibitor H5	Itih5	NA		1.66	0.33	NA		1.92	0.22	NA		1.43	0.28	NA		1.22	0.26
1448162_at	vascular cell adhesion molecule 1	*Vcam1•	21.05	11.97	7.28	3.66	9.53	8.00	13.48	1.62	2.25	0.68	1.69	0.33	8.03	6.45	7.51	3.30
1449009_at	T-cell specific GTPase	Tgtp	1.09	0.19	1.67	2.04	1.11	0.16	2.24	0.26	1.10	0.12	0.90	0.38	1.09	0.27	1.13	0.43
1450767_at	neural precursor cell expressed, developmentally down-regulated gene 9	Nedd9	1.54	0.82	2.14	1.17	1.71	0.47	3.85	3.12	1.20	0.21	2.12	1.24	1.27	0.45	2.43	2.48
1451063_at	syntaxin binding protein 4	Stxbp4	0.89	0.59	1.15	0.40	0.99	0.34	1.37	0.42	0.88	0.10	1.68	0.32	0.79	0.46	1.00	0.27
1453228_at	syntaxin 11	Stx11	NA	3.71	3.63	0.89	NA		5.35	0.97	NA		1.25	0.43	NA	2.39	2.75	0.13
1456063_at	open reading frame 34	ORF34•	1.11	0.61	1.52	0.46	0.93	0.40	1.87	0.11	1.01	0.27	1.56	0.57	0.85	0.41	1.24	0.39
1456773_at	nucleoporin like 2	Nupl2	1.11	0.22	1.95	0.61	1.01	0.26	2.24	0.72	1.06	0.11	1.69	0.43	0.86	0.27	1.55	0.56
1458299_s_at	nuclear factor of kappa light polypeptide gene enhancer in B-cells inhibitor, epsilon	*Nfkbie	4.11	2.44	3.48	0.28	2.84	1.18	6.12	1.46	1.45	0.43	1.32	0.12	2.89	0.55	3.10	1.01
1460447_at	pseudouridylate synthase 7 homolog (S. cerevisiae)-like	Pus7l	1.35	0.29	1.47	0.45	1.30	0.18	1.94	0.34	1.28	0.30	1.73	0.65	1.24	0.32	1.57	0.30

Normalized fold ratio data related to the time matched untreated controls of all present genes were analysed for their cooperation index calculated as I=Fold$_{(TNF\&IL-17)}$ / ((Fold$_{(TNF)}$ + Fold$_{(IL17)}$. Synergism was defined as I>=2, inhibition as I<-2 according to [1]. Standard deviation of the three biological replicates are also indicated.

Reference

1. Zrioual S, Ecochard R, Tournadre A, Lenief V, Cazalis MA, Miossec P: **Genome-wide comparison between IL-17A- and IL-17F-induced effects in human rheumatoid arthritis synoviocytes**. *J Immunol* 2009, **182:**3112-3120.
