# Supplementary material for: Genome-wide comparison between IL-17 and combined TNF-alpha/IL-17 induced genes in primary murine hepatocytes
Source: BMC Genomics. 2010 Apr 7;11:226. doi: 10.1186/1471-2164-11-226 (PMC2858152; doi:10.1186/1471-2164-11-226)
Supplement: Additional file 5 — Gene onthology analysis. Over-representation of GO biological processes in genes upregulated by all stimuli (Table S5) or the subgroup upregulated by IL-1β and TNF-α/IL-17 (Table S7). [file 1471-2164-11-226-S5.PDF]

Additional File 5: Gene onthology analysis

Table S5: Over-pepresentation of GO biological processes in genes upregulated by all stimuli (41 genes).

Property Name	Property Size	Universe Size	Selection Size	p-Value
inflammatory response	94	24084	41	5.45439E-07
intracellular signaling cascade	276	24084	41	0.001224329

Significance of over-representation was estimated using Fisher's Exact Test.

Table S7: Over-representation of GO biological processes in genes upregulated by TNF-α/IL-17 and IL-1β stimulation (77 genes).

Property Name	Property Size	Universe Size	Selection Property Size	Selection Size	p-Value
nucleocytoplasmic transport	10	24084	2	77	0.000447
regulation of transcription from RNA polymerase II promoter	48	24084	3	77	0.000490
cell death	13	24084	2	77	0.000769
calcium-independent cell-cell adhesion	20	24084	2	77	0.001847
acute-phase response	22	24084	2	77	0.002236
cytokine and chemokine mediated signaling pathway	22	24084	2	77	0.002236

Significance of over-representation was estimated using Fisher's Exact Test.
