# Supplementary material for: Genome-wide comparison between IL-17 and combined TNF-alpha/IL-17 induced genes in primary murine hepatocytes
Source: BMC Genomics. 2010 Apr 7;11:226. doi: 10.1186/1471-2164-11-226 (PMC2858152; doi:10.1186/1471-2164-11-226)
Supplement: Additional file 6 — TFBS-overrepresentation analysis. Table S6: Over-representation of transcription factor binding sites in promoters of genes upregulated by all stimuli. [file 1471-2164-11-226-S6.PDF]

Additional file 6: TFBS-over-representation

Table S6: Over-representation of transcription factor binding sites in promoters of genes upregulated by all stimuli (41 genes).

Property Name	PWM	Property Size	Universe Size	Selection Property Size	Selection Size	p-Value
p65	V$NFKAPPAB65_01	1124	24084	12	41	2.26E-07
NF-κB	V$NFKB_Q6	1192	24084	12	41	4.25E-07
p50	V$NFKAPPAB50_01	1342	24084	12	41	1.49E-06
p65	MA0107	1120	24084	11	41	1.83E-06
p50	MA0105	1265	24084	10	41	3.88E-05
NF-κB	V$NFKB_C	1338	24084	10	41	6.24E-05
NF-κB	V$NFKAPPAB_01	1117	24084	9	41	8.79E-05
NF-κB	MA0061	1150	24084	9	41	0.00011
Dorsal_2	MA0023	1214	24084	9	41	0.000166
c-REL	MA0101	1062	24084	8	41	0.000363
c-Rel	V$CREL_01	1062	24084	8	41	0.000363
CCAAT box	V$CAAT_01	1434	24084	9	41	0.000567
repressor of CAR1 expression	F$REPCAR1_01	1489	24084	9	41	0.000744
AP2alpha	MA0003	2910	24084	13	41	0.000754

Significance of over-representation was estimated using Fisher’s Exact Test.
