# Supplementary material for: Genome-wide comparison between IL-17 and combined TNF-alpha/IL-17 induced genes in primary murine hepatocytes
Source: BMC Genomics. 2010 Apr 7;11:226. doi: 10.1186/1471-2164-11-226 (PMC2858152; doi:10.1186/1471-2164-11-226)
Supplement: Additional file 7 — Calculation of the Pearson correlation coefficient to study the "IL-1β like" effect in different gene subsets. Calculation of the Pearson correlation coefficient reveals a shift towards "IL-1β " like expression following combined TNF-α/IL-17 treatment. Contains additional figures: Figure S1: Calculation of the Pearson correlation coefficient to study the "IL-1β like" effect for the 48 probe sets group. Figure S2: Calculation of the Pearson correlation coefficient to study the "IL-1β like" effect for the 89 probe sets group. Figure S3: Calculation of the Pearson correlation coefficient to study the "IL-1β like" effect for those probe sets for which log-ratio data could be calculated (6039 from 14988 probe sets). [file 1471-2164-11-226-S7.PDF]

Additional File 7: Calculation of the Pearson correlation coefficient to study the "IL-1β like" effect for different gene subsets

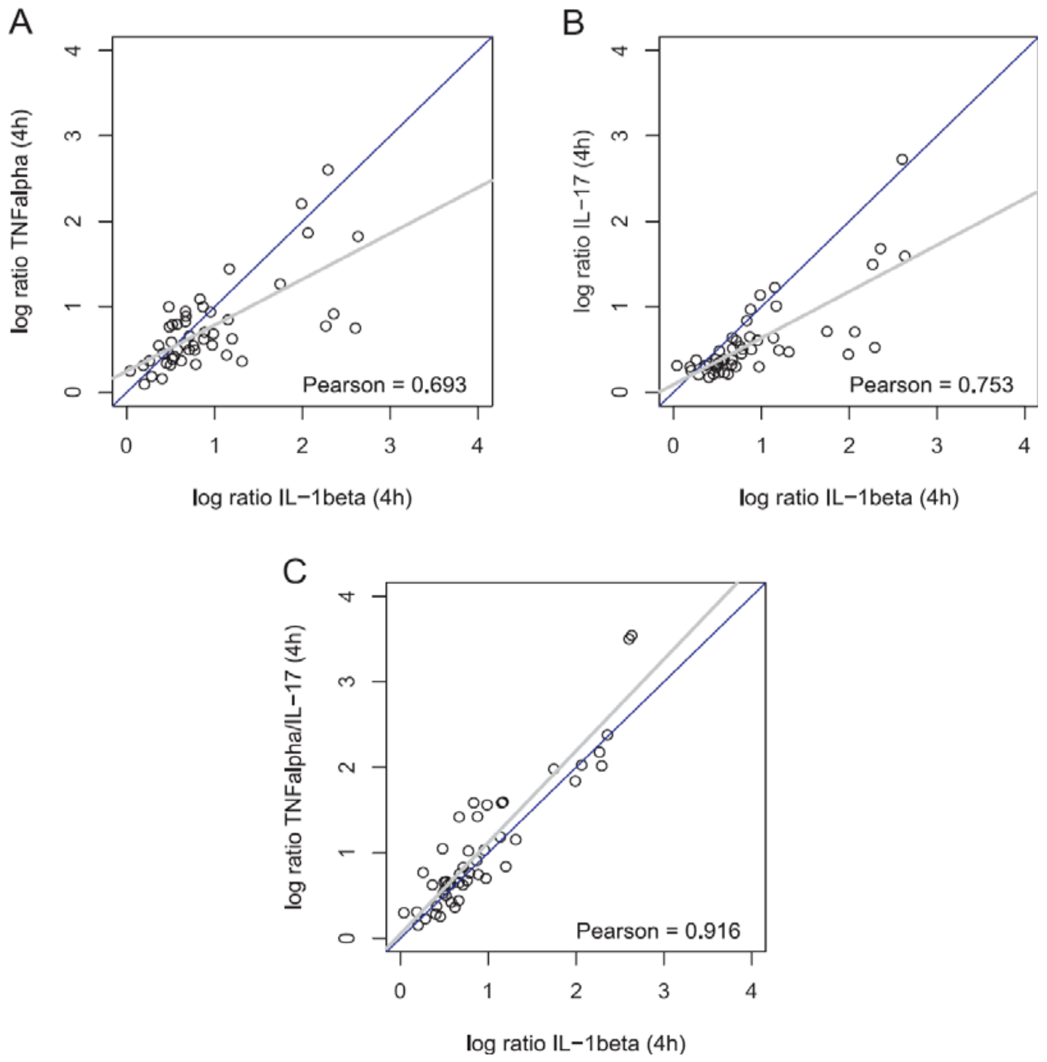

Figure S1: Calculation of the Pearson correlation coefficient to study the "IL-1β like" effect within the gene subgroup "upregulated by all stimuli" (48 probe-sets). Induction ratios of the 48 probe-sets between IL-1β (4h) and the combination of TNF-α/IL-17 were correlated. The X and Y-axis indicate logarithmic fold induction values for the respective stimulation. The blue line indicates a virtual line representing a correlation factor of 1, the grey line the actually calculated regression curve factor for the respective probe sets. The number in the lower right corner reflects the calculated Pearson correlation coefficient.

Combined treatment of TNF-α/IL-17 (C) gives a better correlation to IL-1β induced gene expression compared to single cytokine treatment with TNF-α (A) or IL-17 (B).

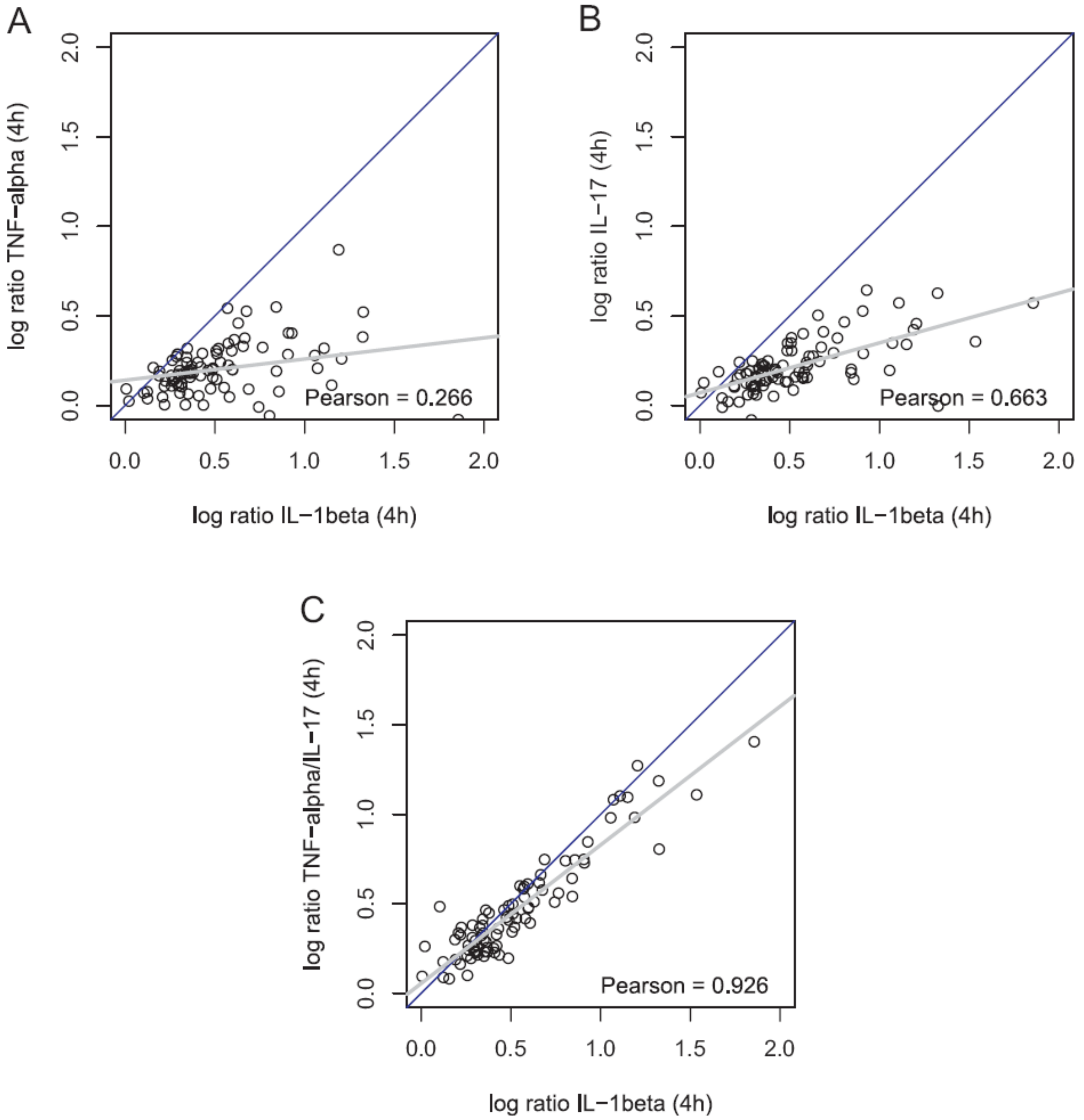

Figure S2: Calculation of the Pearson correlation coefficient to study the "IL-1β like" effect within the IL-17/TNF-α induced gene subset (89 probe-sets). Logarithmic fold induction ratios of the 89 probe-sets between IL-1β (4h) and the combination of TNF-α/IL-17 (C) or between IL-1β and IL-17 (B) or TNF-α (A) were correlated. The X- and Y-axis indicate logarithmic fold induction values for the respective stimulation. The blue line indicates a virtual line representing a correlation factor of 1, the grey line the actually calculated regression curve factor for the respective probe sets.

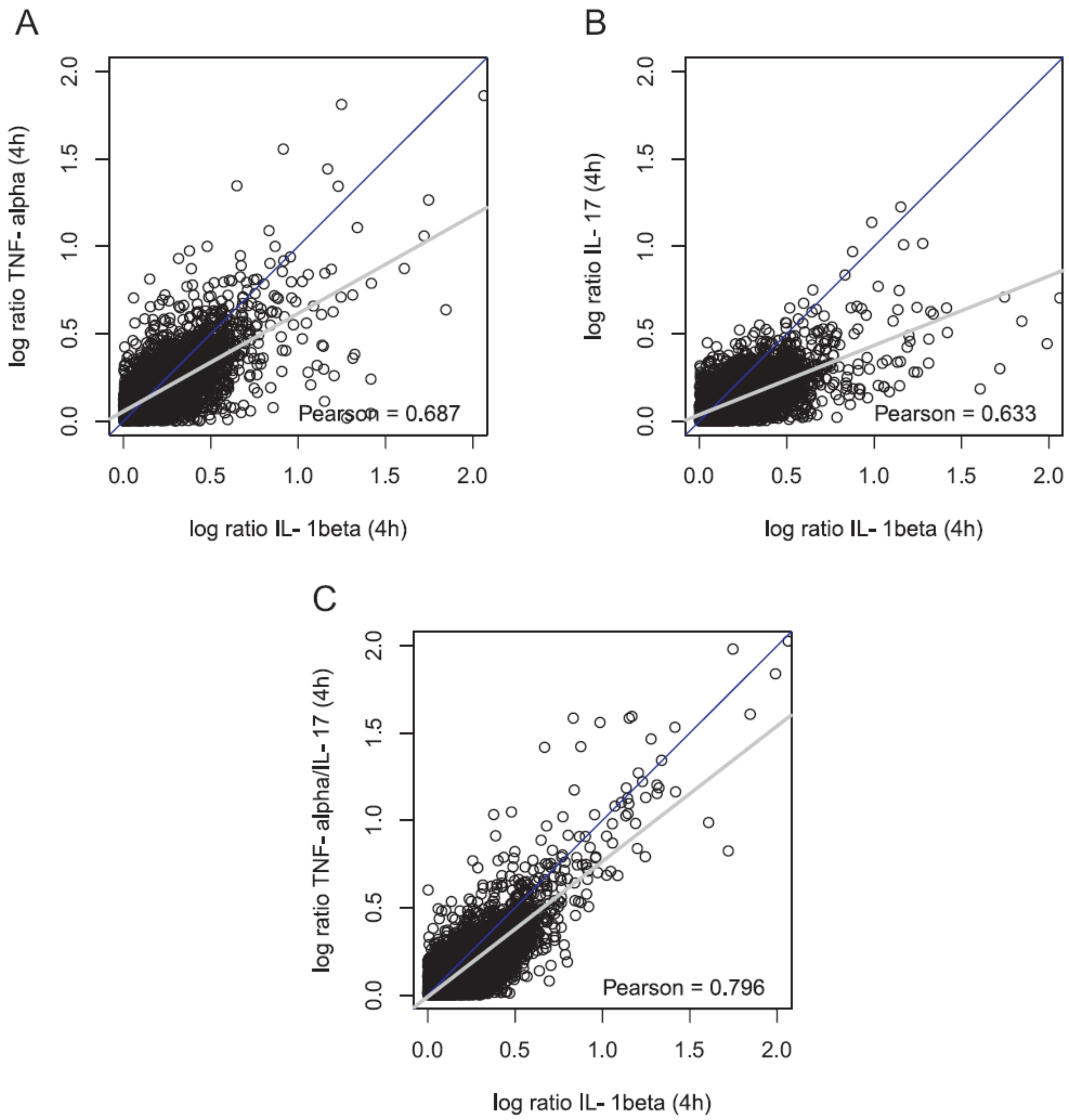

Figure S3: Calculation of the Pearson correlation coefficient to study the "IL-1β like" effect for those probe sets for which log-ratio data could be calculated (6039 from 14988 probe-sets).

Logarithmic fold induction ratios were calculated between IL-1β (4h) and the combination of TNF-α/IL-17 (C) or between IL-1β and IL-17 (B) or TNF-α (A) The X- and Y-axis indicate logarithmic fold induction values for the respective stimulation. The blue line indicates a virtual line representing a correlation factor of 1, the grey line the actually calculated regression curve factor for the respective probe sets.
