# Supplementary material for: Genome-wide comparison between IL-17 and combined TNF-alpha/IL-17 induced genes in primary murine hepatocytes
Source: BMC Genomics. 2010 Apr 7;11:226. doi: 10.1186/1471-2164-11-226 (PMC2858152; doi:10.1186/1471-2164-11-226)
Supplement: Additional file 8 — Time resolved gene expression of Zc3h12a. Figure S4: Time-course of Zc3h12a mRNA expression. [file 1471-2164-11-226-S8.PDF]

Additional file 8: Time-resolved gene expression of Zc3h12a

A

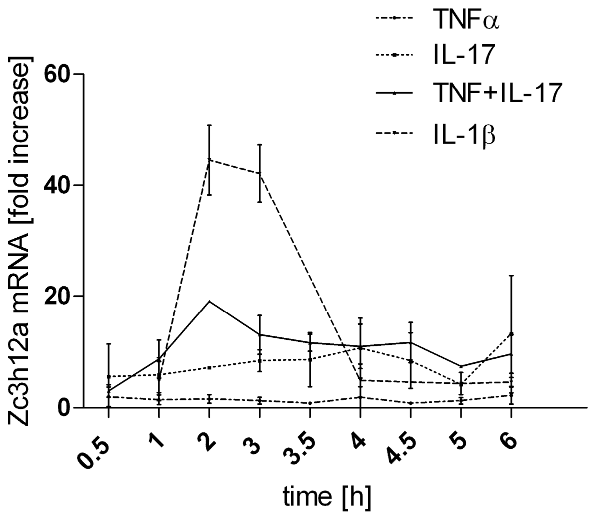

B

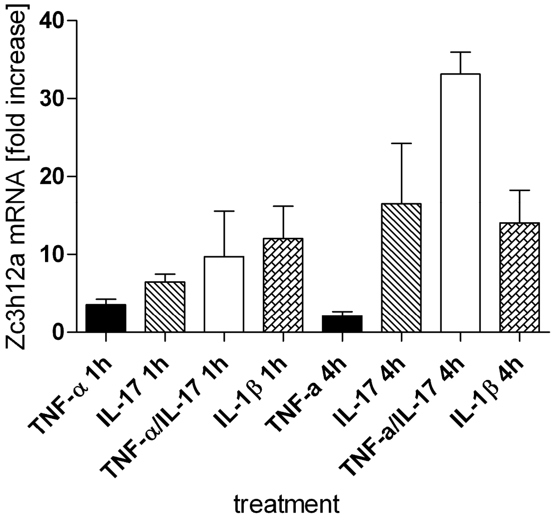

Figure S4: Time-course of Zc3h12a mRNA expression.
(A) Primary hepatocytes were treated with TNF-α (2 ng/ml), IL-17 (100 ng/ml) or the combination of both or with IL-1β (20 ng/ml) and Zc3h12a mRNA expression was determined by qRT-PCR after the indicated times. Data represent means +/- SEM of at least three independent experiments. (B) Zc3h12a mRNA expression levels from the microarray experiment (1h and 4 h). Note, that the expression maximum following IL-1β as well as TNF-α/IL-17 stimulation is located in the range of 1h to 4 h when a time-course was studied by qRT-PCR.
