# Supplementary material for: Genome-wide comparison between IL-17 and combined TNF-alpha/IL-17 induced genes in primary murine hepatocytes
Source: BMC Genomics. 2010 Apr 7;11:226. doi: 10.1186/1471-2164-11-226 (PMC2858152; doi:10.1186/1471-2164-11-226)
Supplement: Additional file 9 — Heatmap of genes upregulated by IL-1β and the combination TNF-α/IL-17. Figure S5: Heatmap of the genes up-regulated by IL-1β and the combination of TNF-α and IL-17 (89 probe sets). [file 1471-2164-11-226-S9.PDF]

Additional file 9: Heatmap of the genes upregulated by IL-1β and the combination of TNF-α/IL-17

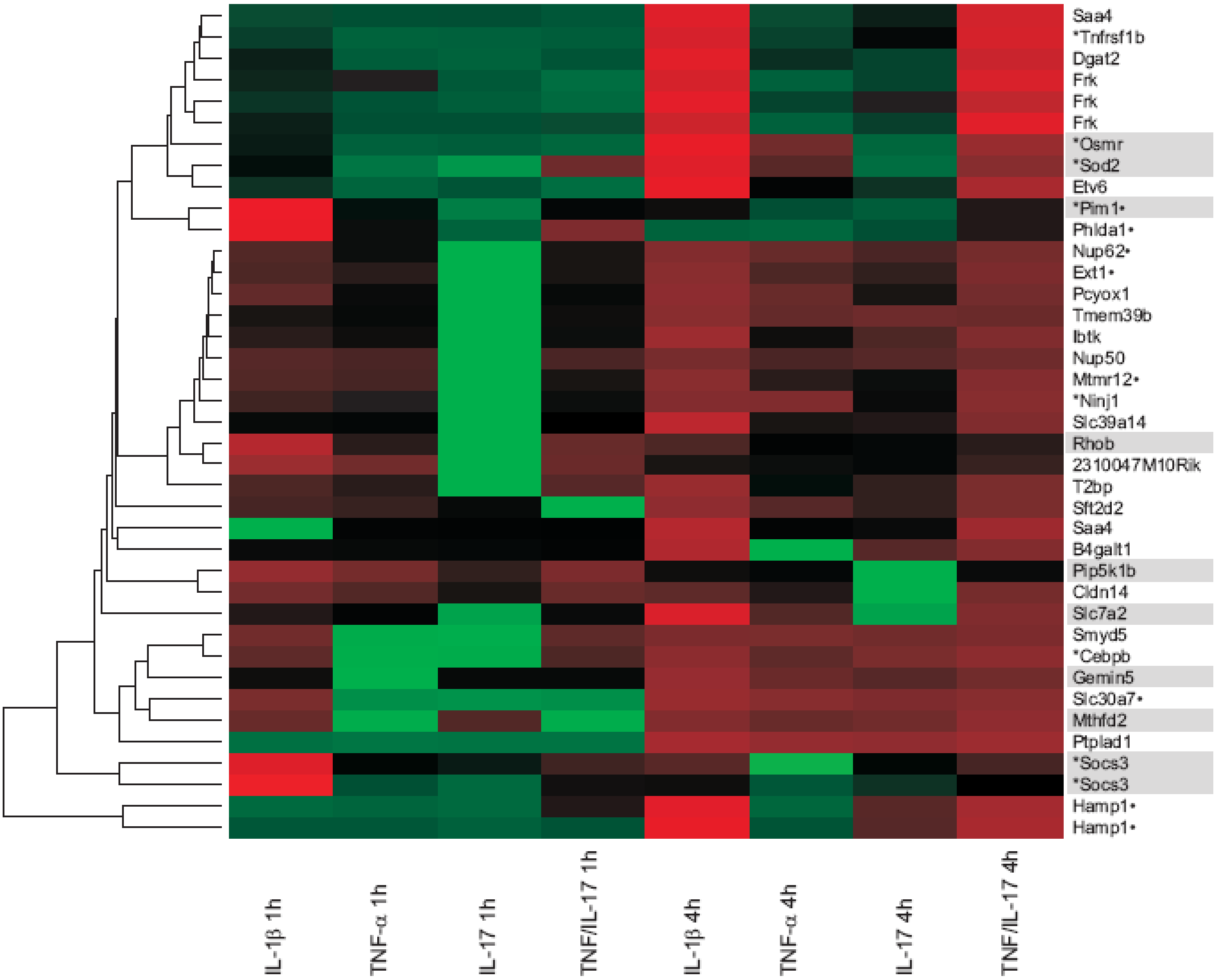

Figure S5: Heatmap of the genes upregulated by IL-1β and the combination of TNF-α and IL-17.

The ANOVA revealed 89 probe-sets which were filtered for a SD >0.4 over all experiments. ARE containing genes are highlighted in grey. NF-κB target genes are indicated by a star (*) for literature derived annotation or a dot (•) for bioinformatical annotation next to the gene symbol.
