# Supplementary material for: Genome-wide comparison between IL-17 and combined TNF-alpha/IL-17 induced genes in primary murine hepatocytes
Source: BMC Genomics. 2010 Apr 7;11:226. doi: 10.1186/1471-2164-11-226 (PMC2858152; doi:10.1186/1471-2164-11-226)
Supplement: Additional file 10 — Hierarchical cluster analysis of genes upregulated by IL-1β, IL-17 and TNF-α/IL-17. Figure S6: Hierarchical cluster analysis of the probe sets (12) upregulated by IL-1β, IL-17, and TNF-α/IL-17, but not TNF-α alone. [file 1471-2164-11-226-S10.PDF]

# Additional file 10: Hierarchical cluster analysis of genes upregulated by IL-1β, IL-17, and TNF-α/IL-17

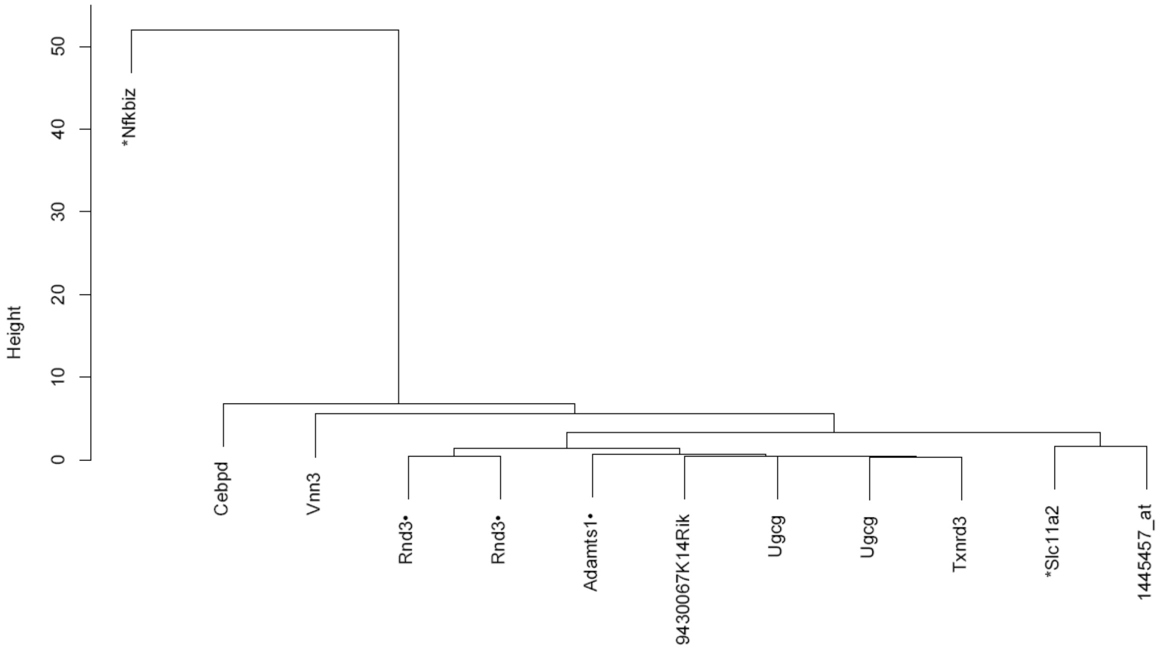


**Figure S6: Cluster analysis of the probe-sets (12) upregulated by IL-1β, IL-17, and TNF-α/IL-17, but not by TNF-α alone.** The genes identified by ANOVA have been hierarchically clustered for similarity of gene expression over all datasets. NF-κB target genes are indicated by *.
